# Supplementary material for: Unsung climate guardians: The overlooked role of remnant and spontaneous trees in carbon stocks and gains from tree growth in West African cocoa fields
Source: PLoS One. 2025 Aug 1;20(8):e0328763. doi: 10.1371/journal.pone.0328763 (PMC12316257; doi:10.1371/journal.pone.0328763)
Supplement: S1 Appendix — (DOCX) [file pone.0328763.s001.docx]

| **Remant** | | **Spontaneous** | | **Planted** | |
| --- | --- | --- | --- | --- | --- |
| **Species** | **family** | **Species** | **family** | Species | **family** |
| ***Acioa barteri* (Hook.f. ex Oliv.) Engl.** | Chrysobalanaceae | ***Aeglopsis chevalieri*** Swingle | Rutaceae | ***Acacia mangium*** Willd. | Fabaceae |
| ***Afrosersalisia afzelii* (Engl.) A.Chev.** | Sapotaceae | ***Afzelia bella*** Harms | Fabaceae | ***Alstonia boonei*** De Wild. | Apocynaceae |
| ***Afzelia bella* Harms** | Fabaceae | ***Aidia genipiflora*** (DC.) Dandy | Rubiaceae | ***Anacardium occidentale*** L. | Anacardiaceae |
| ***Aidia genipiflora* (DC.) Dandy** | Rubiaceae | ***Albizia adianthifolia*** (Schumach.) W.Wight | Fabaceae | ***Annickia polycarpa*** (DC.) Setten & Maas ex I.M.Turner | Annonaceae |
| ***Albizia adianthifolia* (Schumach.) W.Wight** | Fabaceae | ***Albizia ferruginea*** (Guill. & Perr.) Benth. | Fabaceae | ***Annona muricata*** L. | Annonaceae |
| ***Albizia ferruginea* (Guill. & Perr.) Benth.** | Fabaceae | ***Albizia glaberrima*** (Schumach. & Thonn.) Benth. | Fabaceae | ***Antiaris toxicaria*** (J.F.Gmel.) Lesch. | Moraceae |
| ***Albizia glaberrima* (Schumach. & Thonn.) Benth.** | Fabaceae | ***Albizia zygia*** (DC.) J.F.Macbr. | Fabaceae | ***Artocarpus communis*** J.R.Forst. & G.Forst. | Moraceae |
| ***Albizia zygia* (DC.) J.F.Macbr.** | Fabaceae | ***Alchornea cordifolia*** (Schumach.) Müll.Arg. | Euphorbiaceae | ***Azadirachta indica*** A.Juss. | Meliaceae |
| ***Alstonia boonei* De Wild.** | Apocynaceae | ***Alstonia boonei*** De Wild. | Apocynaceae | ***Baphia nitida*** G.Lodd. | Fabaceae |
| ***Amphimas pterocarpoides* Harms** | Fabaceae | ***Amphimas pterocarpoides*** Harms | Fabaceae | ***Beilschmiedia mannii*** (Meisn.) Benth. & Hook.f. ex B.D.Jacks. | Lauraceae |
| ***Anacardium occidentale* L.** | Anacardiaceae | ***Anacardium occidentale*** L. | Anacardiaceae | ***Blighia sapida*** K.D.Koenig | Sapindaceae |
| ***Annickia polycarpa* (DC.) Setten & Maas ex I.M.Turner** | Annonaceae | ***Annickia polycarpa*** (DC.) Setten & Maas ex I.M.Turner | Annonaceae | ***Bombax buonopozense*** P.Beauv. | Malvaceae |
| ***Anthocleista djalonensis* A.Chev.** | Gentianaceae | ***Annona muricata*** L. | Annonaceae | ***Carica papaya*** L. | Caricaceae |
| ***Anthocleista nobilis* G.Don** | Gentianaceae | ***Anthocleista djalonensis*** A.Chev. | Gentianaceae | ***Cassia siamea*** Lam. | Fabaceae |
| ***Anthocleista vogelii* Planch.** | Gentianaceae | ***Anthocleista vogelii*** Planch. | Gentianaceae | ***Cecropia peltata*** L. | Urticaceae |
| ***Anthonotha fragrans* (Baker f.) Exell & Hillc.** | Fabaceae | ***Anthonotha fragrans*** (Baker f.) Exell & Hillc. | Fabaceae | ***Cedrela odorata*** L. | Meliaceae |
| ***Anthonotha macrophylla* P.Beauv.** | Fabaceae | ***Anthonotha macrophylla*** P.Beauv. | Fabaceae | ***Ceiba pentandra*** (L.) Gaertn. | Malvaceae |
| ***Antiaris toxicaria* (J.F.Gmel.) Lesch.** | Moraceae | ***Antiaris toxicaria*** (J.F.Gmel.) Lesch. | Moraceae | *Citrus aurantiifolia* (Christm.) Swingle | Rutaceae |
| ***Antrocaryon micraster* A.Chev. & Guillaumin** | Anacardiaceae | ***Antrocaryon micraster*** A.Chev. & Guillaumin | Anacardiaceae | *Citrus aurantium* L | Rutaceae |
| ***Azadirachta indica* A.Juss.** | Meliaceae | ***Aphania senegalensis*** Radlk. | Sapindaceae | ***Citrus limon*** (L.) Osbeck | Rutaceae |
| ***Baphia bancoensis* Aubrév.** | Fabaceae | ***Artocarpus communis*** J.R.Forst. & G.Forst. | Moraceae | ***Citrus maxima*** (Burm.) Merr. | Rutaceae |
| ***Baphia nitida* G.Lodd.** | Fabaceae | ***Baphia bancoensis*** Aubrév. | Fabaceae | ***Citrus reticulata*** Blanco | Rutaceae |
| ***Baphia pubescens* Hook.f.** | Fabaceae | ***Baphia nitida*** G.Lodd. | Fabaceae | ***Citrus sinensis*** (L.) Osbeck | Rutaceae |
| ***Beilschmiedia mannii* (Meisn.) Benth. & Hook.f. ex B.D.Jacks.** | Lauraceae | ***Baphia pubescens*** Hook.f. | Fabaceae | ***Cocos nucifera*** L. | Arecaceae |
| ***Berlinia confusa* Hoyle** | Fabaceae | ***Beilschmiedia mannii*** (Meisn.) Benth. & Hook.f. ex B.D.Jacks. | Lauraceae | ***Cola nitida*** (Vent.) Schott & Endl. | Malvaceae |
| ***Bersama abyssinica* Fresen.** | Greyiaceae | ***Berlinia grandiflora*** (Vahl) Hutch. & Dalziel | Fabaceae | ***Colura guianensis*** (Nees & Mont.) Trevis. | Lejeuneaceae |
| ***Blighia sapida* K.D.Koenig** | Sapindaceae | ***Blighia sapida*** K.D.Koenig | Sapindaceae | ***Coula edulis*** Baill. | Olacaceae |
| ***Blighia unijugata* Baker** | Sapindaceae | ***Blighia unijugata*** Baker | Sapindaceae | ***Elaeis guineensis*** Jacq. | Arecaceae |
| ***Blighia welwitschii* (Hiern) Radlk.** | Sapindaceae | ***Blighia welwitschii*** (Hiern) Radlk. | Sapindaceae | ***Entandrophragma candollei*** Harms | Meliaceae |
| ***Bombax brevicuspe* Sprague** | Malvaceae | ***Bombax brevicuspe*** Sprague | Malvaceae | ***Eugenia jambos*** L. | Myrtaceae |
| ***Bombax buonopozense* P.Beauv.** | Malvaceae | ***Bombax buonopozense*** P.Beauv. | Malvaceae | ***Ficus exasperata*** Vahl | Moraceae |
| ***Bosqueia angolensis* Ficalho** | Moraceae | ***Bridelia grandis*** Pierre ex Hutch. | Phyllanthaceae | ***Ficus mucuso*** Welw. ex Ficalho | Moraceae |
| ***Bridelia micrantha* (Hochst.) Baill.** | Phyllanthaceae | ***Bridelia micrantha*** (Hochst.) Baill. | Phyllanthaceae | ***Garcinia kola*** Heckel | Clusiaceae |
| ***Bridelia tomentosa* Blume** | Phyllanthaceae | ***Caloncoba gilgiana*** (Sprague) Gilg | Achariaceae | ***Gliricidia sepium*** (Jacq.) Kunth | Fabaceae |
| ***Calpocalyx brevibracteatus* Harms** | Fabaceae | ***Canarium schweinfurtii*** Engl. | Burseraceae | ***Gmelina arborea*** Roxb. ex Sm. | Lamiaceae |
| ***Calpocalyx winkleri* (Harms) Harms** | Fabaceae | ***Carapa procera*** DC. | Meliaceae | ***Guarea cedrata*** Pellegr. ex A.Chev. | Meliaceae |
| ***Canarium schweinfurtii* Engl.** | Burseraceae | ***Carica papaya*** L. | Caricaceae | ***Harungana madagascariensis*** Lam. ex Poir. | Hypericaceae |
| ***Canthium arnoldianum* (De Wild. & T.Durand) Hepper** | Rubiaceae | ***Carpolobia lutea*** G.Don | Polygalaceae | ***Hevea brasiliensis*** (Willd. ex A.Juss.) Müll.Arg. | Euphorbiaceae |
| ***Carapa procera* DC.** | Meliaceae | ***Cassia siamea*** Lam. | Fabaceae | ***Irvingia gabonensis*** (Aubry-Lecomte ex O'Rorke) Baill. | Irvingiaceae |
| ***Carpolobia lutea* G.Don** | Polygalaceae | ***Cecropia peltata*** L. | Urticaceae | ***Jatropha gossypiifolia*** L. | Euphorbiaceae |
| ***Cassia siamea* Lam.** | Fabaceae | ***Ceiba pentandra*** (L.) Gaertn. | Malvaceae | ***Kigelia africana*** (Lam.) Benth. | Bignoniaceae |
| ***Cecropia peltata* L.** | Urticaceae | ***Celtis adolfi-friderici*** Engl. | Cannabaceae | ***Lannea welwitschii*** (Hiern) Engl. | Anacardiaceae |
| ***Ceiba pentandra* (L.) Gaertn.** | Malvaceae | ***Celtis zenkeri*** Engl. | Cannabaceae | ***Mangifera indica*** L. | Anacardiaceae |
| ***Celtis adolfi-friderici* Engl.** | Cannabaceae | ***Chrysophyllum pruniforme*** Engl. | Sapotaceae | ***Mareya micrantha*** Müll.Arg. | Euphorbiaceae |
| ***Celtis zenkeri* Engl.** | Cannabaceae | ***Citrus limon*** (L.) Osbeck | Rutaceae | ***Margaritaria indica*** (Dalzell) Airy Shaw | Phyllanthaceae |
| ***Chrysophyllum albidum* G.Don** | Sapotaceae | ***Citrus maxima*** (Burm.) Merr. | Rutaceae | ***Milicia excelsa*** (Welw.) C.C.Berg | Moraceae |
| ***Chrysophyllum giganteum* A.Chev.** | Sapotaceae | ***Citrus reticulata*** Blanco | Rutaceae | ***Milicia regia*** (A.Chev.) C.C.Berg | Moraceae |
| ***Citrus reticulata* Blanco** | Rutaceae | ***Citrus sinensis*** (L.) Osbeck | Rutaceae | ***Millettia lane-poolei*** Dunn | Fabaceae |
| ***Citrus sinensis* (L.) Osbeck** | Rutaceae | ***Cleistopholis patens*** (Benth.) Engl. & Diels | Annonaceae | ***Morinda lucida*** Benth. | Rubiaceae |
| ***Cleistopholis patens* (Benth.) Engl. & Diels** | Annonaceae | ***Cocos nucifera*** L. | Arecaceae | ***Moringa oleifera*** Lam. | Moringaceae |
| ***Cocos nucifera* L.** | Arecaceae | ***Cola caricifolia*** (G.Don) K.Schum. | Malvaceae | ***Nauclea diderrichii*** (De Wild.) Merr. | Rubiaceae |
| ***Cola caricifolia* (G.Don) K.Schum.** | Malvaceae | ***Cola chlamydantha*** K.Schum. | Malvaceae | ***Nauclea latifolia*** Sm. | Rubiaceae |
| ***Cola chlamydantha* K.Schum.** | Malvaceae | ***Cola gigantea*** A.Chev. | Malvaceae | ***Newbouldia laevis*** (P.Beauv.) Seem. ex Bureau | Bignoniaceae |
| ***Cola gigantea* A.Chev.** | Malvaceae | ***Cola nitida*** (Vent.) Schott & Endl. | Malvaceae | ***Pachira aquatica*** Aubl. | Malvaceae |
| ***Cola nitida* (Vent.) Schott & Endl.** | Malvaceae | ***Coula edulis*** Baill. | Olacaceae | ***Parkia bicolor*** A.Chev. | Fabaceae |
| ***Copaifera salikounda* Heckel** | Fabaceae | ***Crescentia cujete*** L. | Bignoniaceae | ***Parkia biglobosa*** Benth. | Fabaceae |
| ***Cordia millenii* Baker** | Boraginaceae | ***Dacryodes klaineana*** (Pierre) H.J.Lam | Burseraceae | ***Persea americana*** Mill. | Lauraceae |
| ***Cordia senegalensis* Hochst. ex Baker** | Boraginaceae | ***Daniellia thurifera*** Benn. | Fabaceae | ***Petersianthus macrocarpus*** (P.Beauv.) Liben | Lecythidaceae |
| ***Cynometra ananta* Hutch. & Dalziel** | Fabaceae | ***Deinbollia grandifolia*** Hook.f. | Sapindaceae | ***Psidium guajava*** L. | Myrtaceae |
| ***Dacryodes klaineana* (Pierre) H.J.Lam** | Burseraceae | ***Desplatsia chrysochlamys*** (Mildbr. & Burret) Mildbr. & Burret | Malvaceae | ***Ricinodendron heudelotii*** (Baill.) Pierre ex Heckel | Euphorbiaceae |
| ***Daniellia ogea* (Harms) Rolfe ex Holland** | Fabaceae | ***Dialium dinklagei*** Harms | Fabaceae | ***Spondias mombin*** L. | Anacardiaceae |
| ***Deinbollia grandifolia* Hook.f.** | Sapindaceae | ***Dialium guineense*** Willd. | Fabaceae | ***Sterculia tragacantha*** Lindl. | Malvaceae |
| ***Dialium dinklagei* Harms** | Fabaceae | ***Diospyros sanza-minika*** A.Chev. | Ebenaceae | ***Syzygium malaccense*** (L.) Merr. & L.M.Perry | Myrtaceae |
| ***Dialium guineense* Willd.** | Fabaceae | ***Diospyros vignei*** F.White | Ebenaceae | ***Tabernaemontana crassa*** Benth. | Apocynaceae |
| ***Diospyros heudelotii* Hiern** | Ebenaceae | ***Diospyros viridicans*** Hiern | Ebenaceae | ***Tectona grandis*** L.f. | Lamiaceae |
| ***Diospyros mespiliformis* Hochst. ex A.DC.** | Ebenaceae | ***Discoglypremna caloneura*** Prain | Euphorbiaceae | ***Terminalia catappa*** L. | Combretaceae |
| ***Diospyros sanza-minika* A.Chev.** | Ebenaceae | ***Distemonanthus benthamianus*** Baill. | Fabaceae | ***Terminalia ivorensis*** A.Chev. | Combretaceae |
| ***Diospyros vignei* F.White** | Ebenaceae | ***Dracaena mannii*** Baker | Asparagaceae | ***Terminalia superba*** Engl. & Diels | Combretaceae |
| ***Diospyros viridicans* Hiern** | Ebenaceae | ***Ehretia trachyphylla*** C.H.Wright | Boraginaceae | ***Tieghemella heckelii*** (A.Chev.) Pierre ex Dubard | Sapotaceae |
| ***Discoglypremna caloneura* Prain** | Euphorbiaceae | ***Elaeis guineensis*** Jacq. | Arecaceae | ***Tieghemella heckelii*** Roberty | Sapotaceae |
| ***Distemonanthus benthamianus* Baill.** | Fabaceae | ***Entada gigas*** (L.) Fawc. & Rendle | Fabaceae | ***Vernonia colorata*** Drake | Compositae |
| ***Dracaena arborea* (Willd.) Link** | Asparagaceae | ***Entandrophragma angolense*** C.DC. | Meliaceae | ***Vitex rivularis*** Gürke | Lamiaceae |
| ***Dracaena mannii* Baker** | Asparagaceae | ***Entandrophragma cylindricum*** Sprague | Meliaceae | ***Xylopia aethiopica*** (Dunal) A.Rich. | Annonaceae |
| ***Ehretia trachyphylla* C.H.Wright** | Boraginaceae | ***Ficus exasperata*** Vahl | Moraceae |  |  |
| ***Elaeis guineensis* Jacq.** | Arecaceae | ***Ficus glumosa*** Delile | Moraceae |  |  |
| ***Entandrophragma angolense* C.DC.** | Meliaceae | ***Ficus mucuso*** Welw. ex Ficalho | Moraceae |  |  |
| ***Entandrophragma cylindricum* Sprague** | Meliaceae | ***Ficus ottoniifolia*** Miq. | Moraceae |  |  |
| ***Erythrina senegalensis* DC.** | Fabaceae | ***Ficus ovata*** Vahl | Moraceae |  |  |
| ***Erythrina tholloniana* Hua** | Fabaceae | ***Ficus platyphylla*** Delile | Moraceae |  |  |
| ***Erythrina vogelii* Hook.f.** | Fabaceae | ***Ficus populifolia*** Vahl | Moraceae |  |  |
| ***Ficus barteri* Sprague** | Moraceae | ***Ficus sur*** Forssk. | Moraceae |  |  |
| ***Ficus exasperata* Vahl** | Moraceae | ***Ficus variifolia*** Warb. | Moraceae |  |  |
| ***Ficus goliath* A.Chev.** | Moraceae | ***Ficus vogeliana*** Miq. | Moraceae |  |  |
| ***Ficus kamerunensis* Warb. ex Mildbr. & Burret** | Moraceae | ***Funtumia africana*** (Benth.) Stapf | Apocynaceae |  |  |
| ***Ficus mucuso* Welw. ex Ficalho** | Moraceae | ***Funtumia elastica*** (P.Preuss) Stapf | Apocynaceae |  |  |
| ***Ficus populifolia* Vahl** | Moraceae | ***Garcinia kola*** Heckel | Clusiaceae |  |  |
| ***Ficus sur* Forssk.** | Moraceae | ***Glyphaea brevis*** (Spreng.) Monach. | Malvaceae |  |  |
| ***Ficus variifolia* Warb.** | Moraceae | ***Grossera vignei*** Hoyle | Euphorbiaceae |  |  |
| ***Ficus vogeliana* Miq.** | Moraceae | ***Gymnostemon zaizou*** Aubrév. & Pellegr. | Simaroubaceae |  |  |
| ***Funtumia africana* (Benth.) Stapf** | Apocynaceae | ***Hannoa klaineana*** Pierre & Engl. | Simaroubaceae |  |  |
| ***Funtumia elastica* (P.Preuss) Stapf** | Apocynaceae | ***Harrisonia abyssinica*** Oliv. | Rutaceae |  |  |
| ***Garcinia kola* Heckel** | Clusiaceae | ***Harungana madagascariensis*** Lam. ex Poir. | Hypericaceae |  |  |
| ***Gmelina arborea* Roxb. ex Sm.** | Lamiaceae | ***Heritiera utilis*** (Sprague) Sprague | Malvaceae |  |  |
| ***Hannoa klaineana* Pierre & Engl.** | Simaroubaceae | ***Hevea brasiliensis*** (Willd. ex A.Juss.) Müll.Arg. | Euphorbiaceae |  |  |
| ***Harungana madagascariensis* Lam. ex Poir.** | Hypericaceae | ***Holarrhena floribunda*** T.Durand & Schinz | Apocynaceae |  |  |
| ***Holarrhena floribunda* T.Durand & Schinz** | Apocynaceae | ***Homalium letestui*** Pellegr. | Salicaceae |  |  |
| ***Homalium africanum* (Hook.f.) Benth.** | Salicaceae | ***Homalium molle*** Stapf | Salicaceae |  |  |
| ***Homalium letestui* Pellegr.** | Salicaceae | ***Irvingia gabonensis*** (Aubry-Lecomte ex O'Rorke) Baill. | Irvingiaceae |  |  |
| ***Homalium stipulaceum* Welw. ex Mast.** | Salicaceae | ***Khaya grandifoliola*** C.DC. | Meliaceae |  |  |
| ***Irvingia gabonensis* (Aubry-Lecomte ex O'Rorke) Baill.** | Irvingiaceae | ***Khaya ivorensis*** A.Chev. | Meliaceae |  |  |
| ***Keayodendron bridelioides* Leandri** | Phyllanthaceae | ***Kigelia africana*** (Lam.) Benth. | Bignoniaceae |  |  |
| ***Khaya anthotheca* C.DC.** | Meliaceae | ***Klainedoxa gabonensis*** Pierre ex Engl. | Irvingiaceae |  |  |
| ***Khaya grandifoliola* C.DC.** | Meliaceae | ***Lannea nigritana*** (Scott Elliot) Keay | Anacardiaceae |  |  |
| ***Khaya ivorensis* A.Chev.** | Meliaceae | ***Lannea welwitschii*** (Hiern) Engl. | Anacardiaceae |  |  |
| ***Lannea nigritana* (Scott Elliot) Keay** | Anacardiaceae | ***Lophira alata*** Banks ex C.F.Gaertn. | Ochnaceae |  |  |
| ***Lannea welwitschii* (Hiern) Engl.** | Anacardiaceae | ***Macaranga barteri*** Müll.Arg. | Euphorbiaceae |  |  |
| ***Lecaniodiscus cupanioides* Planch.** | Sapindaceae | ***Macaranga hurifolia*** Beille | Euphorbiaceae |  |  |
| ***Lonchocarpus sericeus* (Poir.) Kunth ex DC.** | Fabaceae | ***Maesopsis eminii*** Engl. | Rhamnaceae |  |  |
| ***Macaranga barteri* Müll.Arg.** | Euphorbiaceae | ***Mangifera indica*** L. | Anacardiaceae |  |  |
| ***Magnistipula zenkeri* Engl.** | Chrysobalanaceae | ***Mansonia altissima*** (A.Chev.) A.Chev. | Malvaceae |  |  |
| ***Mangifera indica* L.** | Anacardiaceae | ***Mareya micrantha*** Müll.Arg. | Euphorbiaceae |  |  |
| ***Mansonia altissima* (A.Chev.) A.Chev.** | Malvaceae | ***Margaritaria discoidea*** (Baill.) G.L.Webster | Phyllanthaceae |  |  |
| ***Mareya micrantha* Müll.Arg.** | Euphorbiaceae | ***Milicia excelsa*** (Welw.) C.C.Berg | Moraceae |  |  |
| ***Margaritaria discoidea* (Baill.) G.L.Webster** | Phyllanthaceae | ***Milicia regia*** (A.Chev.) C.C.Berg | Moraceae |  |  |
| ***Memecylon lateriflorum* (G.Don) Bremek.** | Melastomataceae | ***Millettia lane-poolei*** Dunn | Fabaceae |  |  |
| ***Microdesmis keayana* J.Léonard** | Pandaceae | ***Millettia zechiana*** Harms | Fabaceae |  |  |
| ***Milicia excelsa* (Welw.) C.C.Berg** | Moraceae | ***Monodora crispata*** Engl. | Annonaceae |  |  |
| ***Milicia regia* (A.Chev.) C.C.Berg** | Moraceae | ***Monodora myristica*** (Gaertn.) Dunal | Annonaceae |  |  |
| ***Millettia lane-poolei* Dunn** | Fabaceae | ***Morinda lucida*** Benth. | Rubiaceae |  |  |
| ***Millettia zechiana* Harms** | Fabaceae | ***Morus mesozygia*** Stapf | Moraceae |  |  |
| ***Mitragyna ledermannii* (K.Krause) Ridsdale** | Rubiaceae | ***Musanga cecropioides*** R.Br. ex Tedlie | Urticaceae |  |  |
| ***Monodora brevipes* Benth.** | Annonaceae | ***Myrianthus arboreus*** P.Beauv. | Urticaceae |  |  |
| ***Monodora myristica* (Gaertn.) Dunal** | Annonaceae | ***Myrianthus libericus*** Rendle | Urticaceae |  |  |
| ***Monodora tenuifolia* Benth.** | Annonaceae | ***Nauclea diderrichii*** (De Wild.) Merr. | Rubiaceae |  |  |
| ***Morinda lucida* Benth.** | Rubiaceae | ***Nesogordonia papaverifera*** (A.Chev.) Capuron ex N.Hallé | Malvaceae |  |  |
| ***Morus mesozygia* Stapf** | Moraceae | ***Newbouldia laevis*** (P.Beauv.) Seem. ex Bureau | Bignoniaceae |  |  |
| ***Musanga cecropioides* R.Br. ex Tedlie** | Urticaceae | ***Octoknema borealis*** Hutch. & Dalziel | Olacaceae |  |  |
| ***Myrianthus arboreus* P.Beauv.** | Urticaceae | ***Omphalocarpum ahia*** A.Chev. | Sapotaceae |  |  |
| ***Myrianthus libericus* Rendle** | Urticaceae | ***Omphalocarpum elatum*** Miers | Sapotaceae |  |  |
| ***Napoleonaea vogelii* Hook. & Planch.** | Lecythidaceae | ***Pachira aquatica*** Aubl. | Malvaceae |  |  |
| ***Nauclea diderrichii* (De Wild.) Merr.** | Rubiaceae | ***Parkia bicolor*** A.Chev. | Fabaceae |  |  |
| ***Nauclea gilletii* (De Wild.) Merr.** | Rubiaceae | ***Persea americana*** Mill. | Lauraceae |  |  |
| ***Nesogordonia papaverifera* (A.Chev.) Capuron ex N.Hallé** | Malvaceae | ***Petersianthus macrocarpus*** (P.Beauv.) Liben | Lecythidaceae |  |  |
| ***Newbouldia laevis* (P.Beauv.) Seem. ex Bureau** | Bignoniaceae | ***Phyllocosmus africanus*** (Hook.f.) Klotzsch | Ixonanthaceae |  |  |
| ***Octoknema borealis* Hutch. & Dalziel** | Olacaceae | ***Piptadeniastrum africanum*** (Hook.f.) Brenan | Fabaceae |  |  |
| ***Oldfieldia africana* Benth. & Hook.f.** | Picrodendraceae | ***Placodiscus bancoensis*** Aubrév. & Pellegr. | Sapindaceae |  |  |
| ***Ouratea affinis* Engl.** | Ochnaceae | ***Pouteria altissima*** (A.Chev.) Baehni | Sapotaceae |  |  |
| ***Parkia bicolor* A.Chev.** | Fabaceae | ***Psidium guajava*** L. | Myrtaceae |  |  |
| ***Pentaclethra macrophylla* Benth.** | Fabaceae | ***Pterygota bequaertii*** De Wild. | Malvaceae |  |  |
| ***Pentadesma butyracea* Sabine** | Clusiaceae | ***Pterygota macrocarpa*** K.Schum. | Malvaceae |  |  |
| ***Persea americana* Mill.** | Lauraceae | ***Pycnanthus angolensis*** (Welw.) Warb. | Myristicaceae |  |  |
| ***Petersianthus macrocarpus* (P.Beauv.) Liben** | Lecythidaceae | ***Raphia hookeri*** G.Mann & H.Wendl. | Arecaceae |  |  |
| ***Phyllocosmus africanus* (Hook.f.) Klotzsch** | Ixonanthaceae | ***Rauvolfia vomitoria*** Wennberg | Apocynaceae |  |  |
| ***Piptadeniastrum africanum* (Hook.f.) Brenan** | Fabaceae | ***Ricinodendron heudelotii*** (Baill.) Pierre ex Heckel | Euphorbiaceae |  |  |
| ***Placodiscus bancoensis* Aubrév. & Pellegr.** | Sapindaceae | ***Rothmannia hispida*** (K.Schum.) Fagerl. | Rubiaceae |  |  |
| ***Placodiscus pseudostipularis* Radlk** | Sapindaceae | ***Sapium aubrevillei*** Leandri | Euphorbiaceae |  |  |
| ***Pouteria altissima* (A.Chev.) Baehni** | Sapotaceae | ***Scottellia chevalieri*** Chipp | Achariaceae |  |  |
| ***Protomegabaria stapfiana* (Beille) Hutch.** | Phyllanthaceae | ***Scottellia klaineana*** Pierre | Achariaceae |  |  |
| ***Pseudospondias microcarpa* Engl.** | Anacardiaceae | ***Scytopetalum tieghemii*** (A.Chev.) Hutch. & Dalziel | Lecythidaceae |  |  |
| ***Psidium guajava* L.** | Myrtaceae | ***Spathodea campanulata*** P.Beauv. | Bignoniaceae |  |  |
| ***Pterygota macrocarpa* K.Schum.** | Malvaceae | ***Spondianthus preussii*** Engl. | Phyllanthaceae |  |  |
| ***Pycnanthus angolensis* (Welw.) Warb.** | Myristicaceae | ***Spondias mombin*** L. | Anacardiaceae |  |  |
| ***Raphia hookeri* G.Mann & H.Wendl.** | Arecaceae | ***Sterculia rhinopetala*** K.Schum. | Malvaceae |  |  |
| ***Rauvolfia vomitoria* Wennberg** | Apocynaceae | ***Sterculia tragacantha*** Lindl. | Malvaceae |  |  |
| ***Ricinodendron heudelotii* (Baill.) Pierre ex Heckel** | Euphorbiaceae | ***Strombosia pustulata*** Oliv. | Olacaceae |  |  |
| ***Rothmannia whitfieldii* (Lindl.) Dandy** | Rubiaceae | ***Tabernaemontana crassa*** Benth. | Apocynaceae |  |  |
| ***Samanea dinklagei* (Harms) Keay** | Fabaceae | ***Tamarindus indica*** L. | Fabaceae |  |  |
| ***Sapium aubrevillei* Leandri** | Euphorbiaceae | ***Tectona grandis*** L.f. | Lamiaceae |  |  |
| ***Schrebera arborea* A.Chev.** | Oleaceae | ***Terminalia ivorensis*** A.Chev. | Combretaceae |  |  |
| ***Scottellia chevalieri* Chipp** | Achariaceae | ***Terminalia mantaly*** H.Perrier | Combretaceae |  |  |
| ***Scottellia klaineana* Pierre** | Achariaceae | ***Terminalia superba*** Engl. & Diels | Combretaceae |  |  |
| ***Scytopetalum tieghemii* (A.Chev.) Hutch. & Dalziel** | Lecythidaceae | ***Tetrapleura tetraptera*** (Schumach. & Thonn.) Taub. | Fabaceae |  |  |
| ***Spathodea campanulata* P.Beauv.** | Bignoniaceae | ***Tetrorchidium didymostemon*** (Baill.) Pax & K.Hoffm. | Euphorbiaceae |  |  |
| ***Spondias mombin* L.** | Anacardiaceae | ***Treculia africana*** Decne. ex Trécul | Moraceae |  |  |
| ***Sterculia foetida* L.** | Malvaceae | ***Trema guineensis*** (Schum. & Thonn.) Ficalho | Cannabaceae |  |  |
| ***Sterculia oblonga* Mast.** | Malvaceae | ***Trichilia monadelpha*** (Thonn.) [J.J.de](http://J.J.de) Wilde | Meliaceae |  |  |
| ***Sterculia rhinopetala* K.Schum.** | Malvaceae | ***Trichilia tessmannii*** Harms | Meliaceae |  |  |
| ***Sterculia tragacantha* Lindl.** | Malvaceae | ***Triplochiton scleroxylon*** K.Schum. | Malvaceae |  |  |
| ***Strephonema pseudocola* A.Chev.** | Combretaceae | ***Vernonia amygdalina*** Del. | Compositae |  |  |
| ***Strombosia glaucescens* Engl.** | Olacaceae | ***Vernonia colorata*** Drake | Compositae |  |  |
| ***Strombosia pustulata* Oliv.** | Olacaceae | ***Vernonia conferta*** Sch.Bip. ex Baker | Compositae |  |  |
| ***Tabernaemontana crassa* Benth.** | Apocynaceae | ***Vernonia conferta*** Benth. | Compositae |  |  |
| ***Tectona grandis* L.f.** | Lamiaceae | ***Vismia guineensis*** (L.) Choisy | Hypericaceae |  |  |
| ***Terminalia ivorensis* A.Chev.** | Combretaceae | ***Vitex ferruginea*** Schumach. & Thonn. | Lamiaceae |  |  |
| ***Terminalia superba* Engl. & Diels** | Combretaceae | ***Vitex grandifolia*** Gürke | Lamiaceae |  |  |
| ***Tetrapleura tetraptera* (Schumach. & Thonn.) Taub.** | Fabaceae | ***Vitex micrantha*** Gürke | Lamiaceae |  |  |
| ***Tetrorchidium didymostemon* (Baill.) Pax & K.Hoffm.** | Euphorbiaceae | ***Vitex rivularis*** Gürke | Lamiaceae |  |  |
| ***Treculia africana* Decne. ex Trécul** | Moraceae | ***Voacanga africana*** Stapf ex Scott Elliot | Apocynaceae |  |  |
| ***Trema guineensis* (Schum. & Thonn.) Ficalho** | Cannabaceae | ***Xanthium canescens*** Widder | Compositae |  |  |
| ***Trichilia martineaui* Aubrév. & Pellegr.** | Meliaceae | ***Xylopia aethiopica*** (Dunal) A.Rich. | Annonaceae |  |  |
| ***Trichilia megalantha* Harms** | Meliaceae | ***Zanthoxylum gilletii*** (De Wild.) P.G.Waterman | Rutaceae |  |  |
| ***Trichilia monadelpha*** (Thonn.) [J.J.de](http://J.J.de) Wilde | Meliaceae | ***Zanthoxylum leprieurii*** Guill. & Perr. | Rutaceae |  |  |
| ***Trichilia tessmannii* Harms** | Meliaceae |  |  |  |  |
| ***Triplochiton scleroxylon* K.Schum.** | Malvaceae |  |  |  |  |
| ***Uapaca esculenta* A.Chev. ex Aubrév. & Leandri** | Phyllanthaceae |  |  |  |  |
| ***Uapaca guineensis* Müll.Arg.** | Phyllanthaceae |  |  |  |  |
| ***Vernonia colorata* Drake** | Compositae |  |  |  |  |
| ***Vismia guineensis* (L.) Choisy** | Hypericaceae |  |  |  |  |
| ***Vitex micrantha* Gürke** | Lamiaceae |  |  |  |  |
| ***Vitex rivularis* Gürke** | Lamiaceae |  |  |  |  |
| ***Xanthium canescens* Widder** | Compositae |  |  |  |  |
| ***Xylopia aethiopica* (Dunal) A.Rich.** | Annonaceae |  |  |  |  |
| ***Xylopia parviflora* (A.Rich.) Benth.** | Annonaceae |  |  |  |  |
| ***Xylopia quintasii* Pierre ex Engl. & Diels** | Annonaceae |  |  |  |  |
| ***Zanthoxylum gilletii* (De Wild.) P.G.Waterman** | Rutaceae |  |  |  |  |
| ***Zanthoxylum leprieurii* Guill. & Perr.** | Rutaceae |  |  |  |  |
|  |  |  |  |  |  |
|  |  |  |  |  |  |
|  |  |  |  |  |  |
