# Supplementary figures and images for: Unsung climate guardians: The overlooked role of remnant and spontaneous trees in carbon stocks and gains from tree growth in West African cocoa fields
Source: PLoS One. 2025 Aug 1;20(8):e0328763. doi: 10.1371/journal.pone.0328763 (PMC12316257; doi:10.1371/journal.pone.0328763)

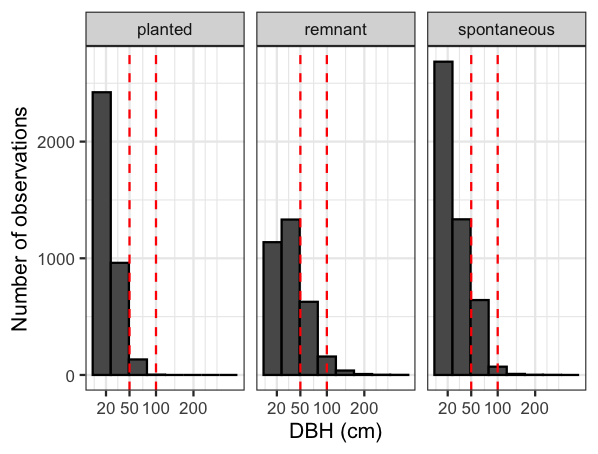

Supplement: S2 Appendix — (TIFF) [file pone.0328763.s002.tiff]

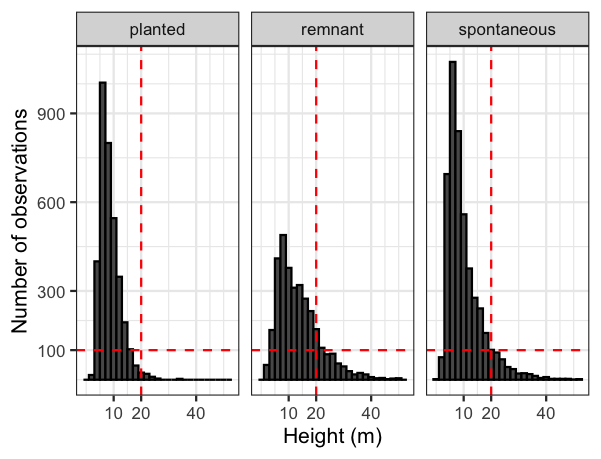

Supplement: S3 Appendix — (TIFF) [file pone.0328763.s003.tiff]

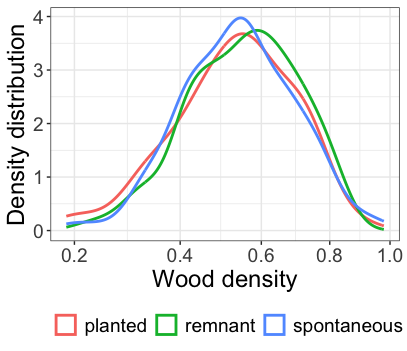

Supplement: S4 Appendix — (TIFF) [file pone.0328763.s004.tiff]
