# Supplementary material for: Unsung climate guardians: The overlooked role of remnant and spontaneous trees in carbon stocks and gains from tree growth in West African cocoa fields
Source: PLoS One. 2025 Aug 1;20(8):e0328763. doi: 10.1371/journal.pone.0328763 (PMC12316257; doi:10.1371/journal.pone.0328763)

PLOS ONE

# Unsung Climate Guardians: The Overlooked Role of Remnant and Spontaneous Trees in Carbon Stocks and Fluxes in West African Cocoa Fields

--Manuscript Draft--

| | |
|---|---|
| **Manuscript Number:** | PONE-D-25-02686 |
| **Article Type:** | Research Article |
| **Full Title:** | Unsung Climate Guardians: The Overlooked Role of Remnant and Spontaneous Trees in Carbon Stocks and Fluxes in West African Cocoa Fields |
| **Short Title:** | Remnant and Spontaneous Trees: Keys to Carbon Storage in Cocoa Fields |
| **Corresponding Author:** | Bruno Hérault<br>CIRAD<br>Montpellier, FRANCE |
| **Keywords:** | Agroforestry; Carbon sequestration; Companion Trees; Growth Modelling; Ivory Coast; Natural regeneration |
| **Abstract:** | Cocoa cultivation in West Africa has driven deforestation, increasing greenhouse gas emissions and threatening cocoa yields, particularly in monoculture systems. Agroforestry, which incorporates trees of different origins—remnant, spontaneous, and planted—offers a sustainable strategy to enhance carbon sequestration and improve farm resilience. However, the specific contributions of these tree origins and the socio-environmental factors influencing their effectiveness are not fully understood. This study investigated carbon dynamics across 150 cocoa fields, encompassing 11,748 trees, in 15 sites in Côte d'Ivoire. Carbon stocks and fluxes were measured, and Bayesian models were used to assess the impact of socio-environmental factors. Carbon stocks varied widely, from 0.19 to 190.11 Mg per hectare, with remnant trees storing the most carbon, followed by spontaneous and planted trees. Spontaneous trees exhibited faster biomass accumulation than planted trees after seven years. Trees of remnant and spontaneous origins are critical in agroforestry systems. Remnant trees store the most carbon and facilitate the regeneration of spontaneous trees, enhancing overall carbon sequestration. Socio-environmental factors, including farmers' botanical knowledge and land ownership, strongly influence carbon stocks and fluxes. Landowners, in particular, tend to conserve spontaneous trees, boosting carbon storage. To optimize carbon sequestration and sustainably manage agroforestry systems, interventions should focus on securing land tenure, providing farmer training in tree botany, and supporting the preservation of remnant and spontaneous trees. These measures are vital for advancing sustainable cocoa production and mitigating climate change. |
| **Order of Authors:** | Isaac Konan Kouamé |
| | Anny Estelle N'Guessan, PhD |
| | Aimé Kouassi |
| | Marie Ruth Dago |
| | Justin Kassi N'Dja, PhD |
| | Raphaël Aussenac, PhD |
| | Stéphane Hubert Guei |
| | Patrick Jagoret, PhD |
| | Bruno Hérault |
| **Additional Information:** | |
| **Question** | **Response** |
| **Financial Disclosure**<br><br>Enter a financial disclosure statement that | Yes |

Powered by Editorial Manager® and ProduXion Manager® from Aries Systems Corporation

| | |
|---|---|
| describes the sources of funding for the work included in this submission. Review the submission guidelines for detailed requirements. View published research articles from *PLOS ONE* for specific examples.<br><br>This statement is required for submission and **will appear in the published article** if the submission is accepted. Please make sure it is accurate.<br><br>**Funded studies**<br>Enter a statement with the following details:<br>• Initials of the authors who received each award<br>• Grant numbers awarded to each author<br>• The full name of each funder<br>• URL of each funder website<br>• Did the sponsors or funders play any role in the study design, data collection and analysis, decision to publish, or preparation of the manuscript?<br><br>Did you receive funding for this work? | |
| Please add funding details.<br>as follow-up to "**Financial Disclosure**<br><br>Enter a financial disclosure statement that describes the sources of funding for the work included in this submission. Review the submission guidelines for detailed requirements. View published research articles from *PLOS ONE* for specific examples.<br><br>This statement is required for submission and **will appear in the published article** if the submission is accepted. Please make sure it is accurate. | This study was conducted within the framework of the Cocoa4Future (C4F) project, which is funded by the European DeSIRA Initiative under grant agreement No. FOOD/2019/412-132 and by the French Development Agency. All authors benefited from this funding. |

| | |
|---|---|
| **Funded studies**<br>Enter a statement with the following details:<br>• Initials of the authors who received each award<br>• Grant numbers awarded to each author<br>• The full name of each funder<br>• URL of each funder website<br>• Did the sponsors or funders play any role in the study design, data collection and analysis, decision to publish, or preparation of the manuscript?<br><br>Did you receive funding for this work?" | |
| Please select the country of your main research funder (please select carefully as in some cases this is used in fee calculation).<br>as follow-up to "**Financial Disclosure**<br><br>Enter a financial disclosure statement that describes the sources of funding for the work included in this submission. Review the submission guidelines for detailed requirements. View published research articles from *PLOS ONE* for specific examples.<br><br>This statement is required for submission and **will appear in the published article** if the submission is accepted. Please make sure it is accurate.<br><br>**Funded studies**<br>Enter a statement with the following details:<br>• Initials of the authors who received each award<br>• Grant numbers awarded to each author<br>• The full name of each funder<br>• URL of each funder website<br>• Did the sponsors or funders play any role in the study design, data collection and analysis, decision to publish, or preparation of the manuscript?<br><br>Did you receive funding for this work?" | BELGIUM - BE |
| **Competing Interests** | The authors have declared that no competing interests exist. |

*Powered by Editorial Manager® and ProduXion Manager® from Aries Systems Corporation*

| | |
|---|---|
| Use the instructions below to enter a competing interest statement for this submission. On behalf of all authors, disclose any competing interests that could be perceived to bias this work—acknowledging all financial support and any other relevant financial or non-financial competing interests.<br><br>This statement is required for submission and **will appear in the published article** if the submission is accepted. Please make sure it is accurate and that any funding sources listed in your Funding Information later in the submission form are also declared in your Financial Disclosure statement.<br><br>View published research articles from *PLOS ONE* for specific examples.<br><br>**NO authors have competing interests**<br><br>Enter: *The authors have declared that no competing interests exist.*<br><br>**Authors with competing interests**<br><br>Enter competing interest details beginning with this statement:<br><br>*I have read the journal's policy and the authors of this manuscript have the following competing interests: [insert competing interests here]*<br><br>* typeset | |
| **Ethics Statement**<br><br>Enter an ethics statement for this submission. This statement is required if the study involved:<br><br>• Human participants<br>• Human specimens or tissue<br>• Vertebrate animals or cephalopods<br>• Vertebrate embryos or tissues | N/A |

| | |
|---|---|
| • Field research<br><br>Write "N/A" if the submission does not require an ethics statement.<br><br>General guidance is provided below. Consult the submission guidelines for detailed instructions. **Make sure that all information entered here is included in the Methods section of the manuscript.** | |

Powered by Editorial Manager® and ProduXion Manager® from Aries Systems Corporation

| | |
|---|---|
| **Format for specific study types**<br><br>**Human Subject Research (involving human participants and/or tissue)**<br>• Give the name of the institutional review board or ethics committee that approved the study<br>• Include the approval number and/or a statement indicating approval of this research<br>• Indicate the form of consent obtained (written/oral) or the reason that consent was not obtained (e.g. the data were analyzed anonymously)<br><br>**Animal Research (involving vertebrate animals, embryos or tissues)**<br>• Provide the name of the Institutional Animal Care and Use Committee (IACUC) or other relevant ethics board that reviewed the study protocol, and indicate whether they approved this research or granted a formal waiver of ethical approval<br>• Include an approval number if one was obtained<br>• If the study involved *non-human primates*, add *additional details* about animal welfare and steps taken to ameliorate suffering<br>• If anesthesia, euthanasia, or any kind of animal sacrifice is part of the study, include briefly which substances and/or methods were applied<br><br>**Field Research**<br><br>Include the following details if this study involves the collection of plant, animal, or other materials from a natural setting:<br>• Field permit number<br>• Name of the institution or relevant body that granted permission | |
| **Data Availability**<br><br>Authors are required to make all data underlying the findings described fully available, without restriction, and from the time of publication. PLOS allows rare exceptions to address legal and ethical concerns. See the PLOS Data Policy and FAQ for detailed information. | Yes - all data are fully available without restriction |

| | |
|---|---|
| A Data Availability Statement describing where the data can be found is required at submission. Your answers to this question constitute the Data Availability Statement and **will be published in the article**, if accepted.<br><br>**Important:** Stating 'data available on request from the author' is not sufficient. If your data are only available upon request, select 'No' for the first question and explain your exceptional situation in the text box.<br><br>Do the authors confirm that all data underlying the findings described in their manuscript are fully available without restriction? | |
| **Describe where the data may be found in full sentences. If you are copying our sample text, replace any instances of XXX with the appropriate details.**<br><br>• If the data are **held or will be held in a public repository**, include URLs, accession numbers or DOIs. If this information will only be available after acceptance, indicate this by ticking the box below. For example: *All XXX files are available from the XXX database (accession number(s) XXX, XXX.).*<br>• If the data are all contained **within the manuscript and/or Supporting Information files**, enter the following: *All relevant data are within the manuscript and its Supporting Information files.*<br>• If neither of these applies but you are able to provide **details of access elsewhere**, with or without limitations, please do so. For example:<br><br>*Data cannot be shared publicly because of [XXX]. Data are available from the XXX Institutional Data Access / Ethics Committee (contact via XXX) for researchers who meet the criteria for access to confidential data.*<br><br>*The data underlying the results presented in the study are available from (include the name of the third party* | All data files are available from the DRYAD database repository (accession number XXX, XXX.) |

Powered by Editorial Manager® and ProduXion Manager® from Aries Systems Corporation

| | |
|---|---|
| *and contact information or URL).*<br>• This text is appropriate if the data are owned by a third party and authors do not have permission to share the data.<br><br>* typeset | |
| Additional data availability information: | Tick here if the URLs/accession numbers/DOIs will be available only after acceptance of the manuscript for publication so that we can ensure their inclusion before publication. |

Powered by Editorial Manager® and ProduXion Manager® from Aries Systems Corporation

Manuscript
Click here to access/download;Manuscript;Article2V2_isk_bh.docx

# Unsung Climate Guardians: The Overlooked Role of Remnant and Spontaneous Trees in Carbon Stocks and Fluxes in West African Cocoa Fields.


## Authors

Isaac Kouamé Konan[1], Anny Estelle N'Guessan[1], Aimé Kouassi[2], Marie Ruth Dago[2], Justin Kassi N'Dja[1], Raphaël Aussenac[2,3,4], Stéphane Guei[5], Patrick Jagoret[6], Bruno Hérault[3,4*]

[1] UPR de Botanique, UFR Bioscience, Université Félix Houphouët-Boigny, Abidjan, Côte d'Ivoire

[2] UMRI SAPT, INPHB, Yamoussoukro, Côte d'Ivoire

[3] UPR Forêts et Sociétés, CIRAD, Montpellier, France

[4] Forêts et Sociétés, Université Montpellier, CIRAD, Montpellier, France

[5] Centre d'excellence Africain sur le changement climatique la biodiversité et l'agriculture durable, Université Félix Houphouët-Boigny, Abidjan, Côte d'Ivoire

[6] CIRAD, UMR ABSys, Montpellier, France

* Corresponding author

E-mail: bruno.herault@cirad.fr

**Abstract**

Cocoa cultivation in West Africa has driven deforestation, increasing greenhouse gas emissions and threatening cocoa yields, particularly in monoculture systems. Agroforestry, which incorporates trees of different origins—remnant, spontaneous, and planted—offers a sustainable strategy to enhance carbon sequestration and improve farm resilience. However, the specific contributions of these tree origins and the socio-environmental factors influencing their effectiveness are not fully understood. This study investigated carbon dynamics across 150 cocoa fields, encompassing 11,568 trees, in 15 sites in Côte d'Ivoire. Carbon stocks and fluxes were measured, and Bayesian models were used to assess the impact of socio-environmental factors. Carbon stocks varied widely, from 0.27 to 146.17 Mg per hectare, with remnant trees storing the most carbon, followed by spontaneous and planted trees. Spontaneous trees exhibited faster biomass accumulation than planted trees after seven years. Trees of remnant and spontaneous origins are critical in agroforestry systems. Remnant trees store the most carbon and facilitate the regeneration of spontaneous trees, enhancing overall carbon sequestration. Socio-environmental factors, including farmers' botanical knowledge and land ownership, strongly influence carbon stocks and fluxes. Landowners, in particular, tend to conserve spontaneous trees, boosting carbon storage. To optimize carbon sequestration and sustainably manage agroforestry systems, interventions should focus on securing land tenure, providing farmer training in tree botany, and supporting the preservation of remnant and spontaneous trees. These measures are vital for advancing sustainable cocoa production and mitigating climate change.

Keywords: Agroforestry, Cocoa farming, tree origin, carbon stock, carbon fluxes, Bayesian modeling

## Introduction

In West Africa, the expansion of agricultural land for cocoa cultivation is a major driver of global greenhouse gas (GHG) emissions, largely due to the widespread deforestation it causes [1-3]. In fact, Vervuurt [4] estimate that approximately 1.47 kg of CO2 is released for every kilogram of cocoa produc[illegible] This deforestation exacerbates climate change, leading to disruptions in global weather patterns, which, in turn, negatively impact agricultural production, including cocoa. As global temperatures rise and precipitation patterns become more unpredictable, the effects on cocoa yields are becoming increasingly severe [5, 6]. This problem is especially critical in areas where cocoa is grown as a monoculture. Without the natural shade provided by diverse vegetation, these monoculture plantations are more vulnerable to the impacts of climate variability [7]. Thus, the interplay between deforestation, GHG emissions, and climate change is creating a feedback loop that threatens not only the environment but also the future of cocoa production in these regions. To address these challenges, agroforestry systems (AFS) have been proposed as a potential solution for both mitigating and adapting to climate change. Several studies [8, 9] have demonstrated that integrating a diversity of species into cocoa plantations through agroforestry can provide ecosystem services that enhance the resilience of cocoa farms. These services include creating a microclimate conducive to cocoa growth [10], improving soil fertility through the provision of nutrients, and reducing soil erosion [11, 12]. In terms of carbon sequestration, agroforestry systems outperform monocultures, with higher carbon storage capacities [10, 13, 14]. Dixon [15] highlighted that when properly managed, AFS can thus act as long-term carbon sinks.

Several international initiatives, such as REDD+ (Reducing Emissions from Deforestation and Forest Degradation), the Cocoa Forest Initiative, and the ARS100 standard in West Africa which was recently proposed and implemented by Côte d'Ivoire and Ghana, aim to promote the adoption of AFS in cocoa production systems [16, 17]. Organizations like Rainforest Alliance, UTZ Certified, and Fairtrade also advocate for agroforestry practices in the cocoa sector [18, 19]. Despite ongoing efforts,

progress has been hindered by an overemphasis on only tree planting, often at the expense of promoting natural regeneration [20]. Recent studies [20, 21] have indeed highlighted the coexistence of three distinct types of tree cohorts within cocoa AFS: (1) remnant trees, preserved during land clearing; (2) spontaneous trees, which regenerate naturally during or after cocoa field establishment; and (3) planted trees, intentionally introduced by farmers. Preliminary evidence suggests that remnant and spontaneous trees could have higher species diversity and potentially greater carbon sequestration capacity compared to planted trees [20, 22]. However, despite the increasing interest in carbon stocks and fluxes in cocoa plantations, research remains limited due to the influence of various socio-environmental factors [9, 23]:

- Climatic conditions, particularly precipitation and temperature, are crucial for carbon dynamics in cocoa plantations. Adequate rainfall supports tree growth and photosynthesis, while insufficient precipitation can stunt growth and reduce carbon uptake [24-26]. Excessive rainfall may lead to soil erosion and nutrient loss. Additionally, higher temperatures can slow carbon accumulation due to trees' physiological responses that limit evapotranspiration [27, 28].
- Social factors, including farmers' knowledge of the environment and land ownership, influence carbon storage. Farmers with a strong understanding of local ecosystems are more likely to implement effective management practices that enhance carbon dynamics [21, 29, 30]. Non-owner farmers might prioritize short-term gains, impacting their long-term sustainability practices.
- Field characteristics, such as cocoa planting density and previous land use, also affect carbon dynamics. High planting density increases competition with associated trees, limiting biomass and carbon sequestration [10, 31]. In contrast, lower density allows for greater biodiversity and carbon storage. The previous land use is also significant; fields converted from forests typically retain higher carbon stocks compared to those converted from other agricultural uses [9, 32].

- Soil characteristics, particularly organic matter and bulk density, are vital for aboveground carbon storage potential. High organic matter content enhances soil health and carbon sequestration in trees [33]. Conversely, high bulk density indicates soil compaction, which restricts root growth and limits the soil's ability to retain water and nutrients. Healthy soils not only support robust tree growth but also enhance the ability of these systems to sequester carbon [34].

To date, no comprehensive study has examined the combined impact of socio-environmental factors on carbon stocks and fluxes in trees within cocoa agroforestry systems. To fill this gap, the present study aims to identify and assess the factors influencing the carbon sequestration capacity of trees in cocoa farms, with the goal of enhancing their role as sustainable terrestrial carbon sinks. Specifically, this research seeks to address the following questions: (1) What are the carbon stock levels of planted, spontaneous, and remnant trees in cocoa farms? (2) What is the relative contribution of these tree cohorts to carbon fluxes within cocoa farms? (3) What are the key socio-environmental factors influencing carbon stocks and fluxes in these systems?

## 1. Materials and Methods

### 1.1. Study Sites

Study plots have been selected from 15 locations throughout the cocoa production area (the forest zone) of Côte d’Ivoire (Fig. 1). These sites are situated along a South-North climate gradient, with mean annual temperatures ranging from 22.6°C to 26.2°C and annual rainfall varying between 1,900 mm and 1,100 mm. This climate gradient corresponds to a South-North vegetation gradient, transitioning from evergreen forests to semi-deciduous forests. To capture the diversity of cocoa fields, we identified 10 cocoa fields at each site, focusing on three key criteria: (i) structural complexity, ranging from nearly full-sun cocoa monocultures to complex cocoa agroforests; (ii) age of the cocoa fields, encompassing both young (< 20 years) and mature (> 20 years) plots; and (iii) cocoa tree yield, varying from low productivity (< 300 kg) to high productivity (> 300 kg). The study plots vary in size

from 0.3 to 5 hectares and serve as the sampling units, corresponding to the management units of the farmers.

**Fig 1. Map of the 15 study sites illustrating the diversity of forest types and annual precipitation across Ivory Coast, West Africa.**

### 1.2 Data Collection

All cocoa fields were mapped using GPS technology for precise delineation. Each associated tree with a minimum diameter at breast height (dbh) of 10 cm was identified to the species level. The dbh and the height of each tree was recorded. The origin of each tree—classified as planted, remnant, or spontaneous— and the age of each planted and spontaneous tree was recorded based on the farmer's declaration.

Eight socio-environmental factors of interest were identified for this study (Table 1):

- Two social factors related to the cocoa farmer: botanical knowledge and land ownership status of the cocoa field, obtained through interviews with the farmers.
- Two soil factors: organic carbon content and bulk density of the soil, compiled from existing databases (https://soilgrids.org/).
- Two field-related factors: cocoa tree density and previous land use. Cocoa tree density was estimated by counting the number of cocoa trees in a 1000 m² area within each cocoa field, and previous land use was obtained through interviews with the farmers.
- Two climate factors: annual temperature and precipitation, compiled from existing databases (https://chelsa-climate.org/).

Table 1 – list of the 8 socio-environmental factors studied to explain the variability of carbon stocks and fluxes in the 150 cocoa fields.

| | **Name** | **Description** | **Unit** | **Source** |
|---|---|---|---|---|
| **Social** | Farmers ownership | the ownership status of the farmer's cocoa (owner or not owner) | Binary | Interviews with farmers |
| | Farmers's tree knowledge | Recognition test of the 23 most common species in agroforestry | % | Interviews with farmers |
| **Field** | Precursor landscape | Previous landscape (forest or not) | Binary | Interviews with farmers |
| | Cocoa density | Cocoa tree density | Ind/ha | Measured on 1000 m2 square |
| **Soil** | Bulk density | Bulk density from a depth of 30 to 60 cm | $Kg/dm^3$ | https://soilgrids.org/ |
| | Soil organic carbon content | Soil organic carbon content (fine soil fraction) | g/kg | https://soilgrids.org/ |
| **Climate** | Annual temperature | Annual temperature from 1979 to 2013 at 1 km resolution | Deg C | https://chelsa-climate.org/ |
| | Annual precipitation | Annual precipitation from 1979 to 2013 at 1 km resolution | mm | https://chelsa-climate.org/ |

## 1.3. Data Analysis

The aboveground biomass of each individual tree was estimated and then converted to Carbon, using the computeAGB() function from the BIOMASS package [35, 36] in the R software. We evaluated the effects of cohorts (planted, remnant, spontaneous) and 8 socio-environmental *se* factors on (i) Carbon stock (at the plot scale) and (ii) Carbon flux (at the individual scale, considering that each individual has a different age) using lognormal likelihood models. This likelihood was chosen because the response variables are defined on R+.

Carbon stocks were modelled with the following equation

$$Cs_{coh,p} \sim LN[log(\theta_{coh} \times e^{(\sum_{se=1}^{8}(\theta_X \times X_{se,p}))}), \sigma^2_{Cs}]$$

Where $Cs_{coh,p}$ is the observed Carbon stock (Mg) of cohort *coh* in plot $p$, $\theta_{coh}$ is the predicted average carbon stock (Mg) of cohort *coh* in average environmental conditions (*i.e.* when $X_{se,p}$ equal zero), $\theta_X$ are the standardized (*i.e.* centred and reduced) effects of the socio-environmental factors and $X_{se,p}$ are the observed values of the socio-environmental factors *se* in a given plot *p*. The effects of the socio-environmental factors are included into an exponential function to guarantee positive predictions.

Given that the evolution of the tree carbon over time is well approximated by a power law because growth rates scale with the tree's size, following geometric and metabolic constraints [37], carbon fluxes in time were modelled with the following equation

$$Cf_{i,sp,coh,p} \sim LN[log(\theta_{coh} \times \theta_{sp} \times Age_i^{\beta_{coh}} \times e^{(\sum_{se=1}^{8}(\theta_X \times X_{se,p}))}), \sigma^2_{Cf}]$$

$$\theta_{sp} \sim LN(log(1), \sigma^2_{sp})$$

Where $Cf_{i,sp,coh,p}$ is the observed carbon of tree *i* from cohort *coh* of species *sp* in plot $p$, $\theta_{coh}$ is the effect of cohort on carbon flux (kg.year$^{-1}$), $\theta_{sp}$ is the effect of species on carbon flux with a prior

distribution lognormal centred on 0, $\beta_{coh}$ is, for a given cohort $coh$, the power law coefficient, $\theta_X$ are the standardized (*i.e.* centred and reduced) effects of the socio-environmental factors and $X_{se,p}$ are the observed values of the socio-environmental factors *se* in a given plot $p$. The effects of the socio-environmental factor are included into an exponential function to guarantee positive predictions. Note that because the age of remnant trees is, by definition, unknown, the carbon flux model was inferred for planted and spontaneous trees only.

All models were implemented in R and inferred within a Bayesian framework using Stan, which relies on the Hamiltonian Monte Carlo (HMC) method to achieve full Bayesian inference [38].

## 2. Results

A total of 11,568 trees were inventoried. Spontaneous trees were the most represented cohort, with 4,734 individuals (41%), followed by planted trees with 3,520 individuals (30%), and finally remnant trees with 3,304 individuals (28%).The remnant tree cohort naturally includes a higher proportion of large trees, with 25% having diameters exceeding 50 cm and 66% reaching heights over 25 m. In contrast, the spontaneous tree cohort comprises 15% of trees with diameters greater than 50 cm and 45% with heights exceeding 25 m. The planted tree cohort contains a very small proportion of large trees, with only 4% having diameters over 50 cm and 32% reaching heights greater than 25 m.

### 2.1. Carbon Stocks

Carbon stocks per hectare range from 0.27 to 146.17 Mg, with a median of 14.68 Mg. The carbon stock values by cohort reveal higher carbon stocks for remnant trees, in contrast to the lower stocks observed in planted and spontaneous trees (Fig 2). For remnant trees, carbon stocks per plot range from 0.01 to 92.27 Mg/ha, with a median of 6.74 Mg/ha. Meanwhile, carbon stocks for spontaneous and planted trees range from 0.0034 to 146.17 Mg/ha, with a median of 2.19 Mg/ha, and from 0.03 to 14.11 Mg/ha, with a median of 1.63 Mg/ha, respectively.

**Fig 2. The distribution of carbon stocks across 150 cocoa fields is shown, with carbon stock on the x-axis and the density of values on the y-axis.**

### 2.2. Carbon Fluxes

The carbon accumulation curves show significant differences between planted and spontaneous trees (Fig 3). Biomass accumulation for both tree types is almost identical between 1 and 7 years, with an average accumulation of 6,8 ± 0,89 kgC and 8,9 ± 0,9 kgC per year respectively for planted and spontaneous trees. However, the accumulation diverges after 7 years, with planted trees accumulating biomass more slowly (3.8 ± 0,6 kgC per year) while spontaneous tree has 14,4 ± 1,7 kgC per year. At 40 years, an average spontaneous tree will have a carbon stock of 471.2 kgC, while a planted tree will only have 200.98 kgC. Therefore, the difference in carbon flux is substantial and increases with age for spontaneous and decreases for plantes trees.

**Fig 3. Carbon accumulation of planted and spontaneous trees.** The points represent the observed carbon stock of trees, while the lines show the predicted temporal dynamics of carbon stock (a) and flux (b), with the 95% credibility intervals shaded. This figure is represented on a logarithmic scale

### 2.3. Effects of socio-environmental factors

Carbon stock values of the studied plots are mainly influenced by the botanical knowledge of the cocoa farmer, the ownership status of the cocoa farmer, and the cocoa tree density (Fig 4). These are the factors with the most absolute parameter values different from zero after model inference. Being the owner of the plot ($\theta = -0.44 \pm 0.1$) and having plots with high cocoa tree density ($\theta = -0.34 \pm 0.09$) have negative effects on carbon stock, while the cocoa farmer's botanical knowledge level ($\theta = 0.15 \pm 0.08$) has a positive effect on the tree carbon stock of cocoa plantations.

Carbon flux at the tree level within cocoa plantations is primarily influenced by the cocoa farmer's ownership status, the previous land-use, and annual temperature (Fig 5). Both the ownership of the

plot by the cocoa farmer and the previous land-use (when it's forest) have a positive effect on carbon sequestration by individual trees (θ = 0.19 ± 0.04 and θ = 0.13 ± 0.03, respectively). In contrast, both annual temperature and cocoa density negatively affect carbon sequestration at the tree level, with estimated parameters of θ = -0.8 ± 0.01 and θ = -0.13 ± 0.01, respectively.

**Fig 4. Effect of social, field, soil, and climate factors on plot-level carbon stock.** All covariates were standardized, allowing for direct comparison of their relative effects.

**Fig 5. Effect of soil, field, social, and climate factors on tree-level carbon flux.** All covariates were standardized, enabling direct comparison of their relative effects.

## 3. Discussion

This study helps better understand the carbon sequestration mechanisms in cocoa-based agroforestry systems, and thus provides a foundation for developing management strategies to combat climate change. The objective of this study was to identify the sources of carbon stock and flux in trees within cocoa fields in West Africa. The carbon stock in these fields is primarily derived from remnant trees, while the stocks from planted and spontaneous trees are much lower. In contrast, for carbon flux, cocoa fields accumulate more carbon through spontaneous trees than through planted trees. Several socio-environmental factors explain the levels of carbon stock and flux for each tree cohort. We note that the carbon stock and flux results would be lower because the allometric equation used is calibrated for species in forest landscapes. Therefore, there would be an overestimation of the carbon stock and flux of planted and spontaneous species. However, this has no impact on carbon stock and flux levels by cohort.

### 3.1. Carbon Giants : The Remnants

Our results indicate that the carbon stock in cocoa agroforestry systems (CAFs) is closely linked to remnant trees (Fig 2). Studies by Andreotti [39], Bastin [40], and Sanial [21] have also shown that these remnant trees store more carbon than the other cohorts. Remnants are trees that survived the attempted destruction, often spared or left intact by cocoa farmers during deforestation [41]. As such, they are of forest origin and include species such as *Milicia excelsa (Welw.) Benth, Alstonia boonei De Wild, Amphimas pterocarpoides Harms* and *Petersianthus macrocarpus (P. Beauv.) Liben* (Table 2). These remnant trees, typically large in diameter and height [21], have a higher carbon storage potential, as shown in Table 2. Indeed, tree size (height and diameter) directly determines carbon stock: the larger and thicker the tree, the more carbon it stores [22]. In addition to size, species diversity also plays a crucial role in carbon storage. Greater diversity promotes higher carbon stock [22, 32, 42]. Among the three cohorts studied, the remnant cohort has the highest species diversity [21, 43], which may contribute to greater carbon storage in cocoa plantations. In contrast, the planted tree cohort, mainly consisting of small fruit tree species with low diameters, stores the least carbon. These trees include species such as *Citrus sinensis (L.) Osbeck, Psidium guajava Linn,* and *Anacardium occidentale Linn* (Table 2). The ownership status of the cocoa farmer is one of the most significant factors influencing the carbon stock in cocoa fields. A lower carbon stock is observed when the field is managed by the owner, whereas a higher carbon stock is found in fields managed by non-owners. Cocoa farmers who own the land have full rights to manage it, allowing them to prioritize short-term economic gains from cocoa cultivation [44, 45]. With the freedom to make decisions, they are more likely to remove large associated trees to maximize space and resources for cocoa trees (indeed, cocoa density is also negatively linked to tree carbon stock, Fig 4), which they view as the primary source of income [29]. This ownership gives them the incentive to clear trees that may be seen as competing for nutrients, water, and light. In contrast, non-owners, such as sharecroppers or tenants, have limited rights to modify the land and typically may not have the authority to remove trees, especially if doing so requires the landowner's approval. Non-owners may also value more the ecological benefits of large trees [29], which could discourage them from clearing the land.

Table 2 : Top 30 Species by Carbon Stocks for Each Tree Origin (Remnant, Spontaneous, and Planted) in the 150 Studied Fields (240.5 ha)

| Remnants | | | | Spontaneous | | | | Planted | | | |
|---|---|---|---|---|---|---|---|---|---|---|---|
| **Species** | **Family** | **N** | **Total carbon stock (Mg)** | **Species** | **Family** | **N** | **Total carbon stock (Mg)** | **Species** | **Family** | **N** | **Total carbon stock (Mg)** |
| *Ceiba pentandra* | Malvaceae | 99 | 428.6 | *Elaeis guineensis* | Arecaceae | 993 | 248.6 | *Persea americana* | Lauraceae | 852 | 133.7 |
| *Milicia excelsa* | Moraceae | 85 | 224.8 | *Ceiba pentandra* | Malvaceae | 47 | 90.2 | *Mangifera indica* | Anacardiaceae | 377 | 129.3 |
| *Alstonia boonei* | Apocynaceae | 110 | 138.1 | *Alstonia boonei* | Apocynaceae | 58 | 85.2 | *Cola nitida* | Malvaceae | 321 | 62.1 |
| *Bombax buonopozense* | Malvaceae | 44 | 113.8 | *Pycnanthus angolensis* | Myristicaceae | 84 | 57.0 | *Citrus sinensis* | Rutaceae | 602 | 35.5 |
| *Pycnanthus angolensis* | Myristicaceae | 81 | 103.1 | *Ricinodendron heudelotii* | Euphorbiaceae | 49 | 47.4 | *Hevea brasiliensis* | Euphorbiaceae | 290 | 33.5 |
| *Elaeis guineensis* | Arecaceae | 229 | 99.1 | *Morinda lucida* | Rubiaceae | 144 | 46.7 | *Terminalia superba* | Combretaceae | 120 | 22.5 |
| *Antiaris toxicaria* | Moraceae | 69 | 81.9 | *Albizia zygia* | Fabaceae | 98 | 42.7 | *Elaeis guineensis* | Arecaceae | 51 | 17.1 |
| *Cola gigantea* | Malvaceae | 29 | 70.6 | *Amphimas pterocarpoides* | Fabaceae | 63 | 42.1 | *Cocos nucifera* | Arecaceae | 138 | 16.0 |
| *Ricinodendron heudelotii* | Euphorbiaceae | 62 | 58.4 | *Spathodea campanulata* | Bignoniaceae | 39 | 41.5 | *Tectona grandis* | Lamiaceae | 95 | 13.5 |
| *Amphimas pterocarpoides* | Fabaceae | 64 | 58.2 | *Milicia excelsa* | Moraceae | 69 | 39.4 | *Petersianthus macrocarpus* | Lecythidaceae | 1 | 6.4 |
| *Entandrophragma angolense* | Meliaceae | 57 | 42.5 | *Antiaris toxicaria* | Moraceae | 95 | 37.1 | *Terminalia ivorensis* | Combretaceae | 14 | 5.1 |
| *Albizia adianthifolia* | Fabaceae | 52 | 41.2 | *Sterculia tragacantha* | Malvaceae | 71 | 34.8 | *Garcinia kola* | Clusiaceae | 6 | 4.6 |
| *Mangifera indica* | Anacardiaceae | 76 | 40.1 | *Blighia sapida* | Sapindaceae | 7 | 34.3 | *Anacardium occidentale* | Anacardiaceae | 188 | 4.5 |
| *Terminalia superba* | Combretaceae | 47 | 38.9 | *Cecropia peltata* | Urticaceae | 124 | 32.6 | *Spondias mombin* | Anacardiaceae | 32 | 4.2 |
| *Morinda lucida* | Rubiaceae | 96 | 38.8 | *Ficus exasperata* | Moraceae | 297 | 28.3 | *Gmelina arborea* | Lamiaceae | 14 | 3.4 |
| *Petersianthus macrocarpus* | Lecythidaceae | 15 | 37.4 | *Terminalia superba* | Combretaceae | 82 | 27.3 | *Citrus muricata* | Rutaceae | 44 | 3.4 |
| *Hannoa klaineana* | Simaroubaceae | 31 | 36.0 | *Discoglypremna caloneura* | Euphorbiaceae | 40 | 23.8 | *Milicia excelsa* | Moraceae | 9 | 3.3 |
| *Cola nitida* | Malvaceae | 136 | 35.8 | *Zanthoxylum gilletii* | Rutaceae | 13 | 23.4 | *Citrus maxima* | Rutaceae | 42 | 3.2 |
| *Piptadeniastrum africanum* | Fabaceae | 23 | 33.2 | *Lannea welwitschii* | Anacardiaceae | 44 | 23.2 | *Cedrela odorata* | Meliaceae | 10 | 2.9 |
| *Albizia zygia* | Fabaceae | 69 | 31.1 | *Mangifera indica* | Anacardiaceae | 88 | 22.5 | *Psidium guajava* | Myrtaceae | 55 | 2.7 |
| *Sterculia tragacantha* | Malvaceae | 64 | 29.9 | *Petersianthus macrocarpus* | Lecythidaceae | 23 | 22.0 | *Artocarpus communis* | Moraceae | 15 | 2.6 |
| *Zanthoxylum gilletii* | Rutaceae | 29 | 28.8 | *Bombax buonopozense* | Malvaceae | 19 | 19.1 | *Tieghemella heckelii* | Sapotaceae | 1 | 2.4 |
| *Piptadeniastrum africana* | Fabaceae | 7 | 23.3 | *Cola nitida* | Malvaceae | 84 | 18.2 | *Ricinodendron heudelotii* | Euphorbiaceae | 19 | 2.4 |
| *Bombax brevicuspe* | Malvaceae | 8 | 23.0 | *Strombosia pustulata* | Olacaceae | 18 | 18.0 | *Irvingia gabonensis* | Irvingiaceae | 10 | 2.3 |

| | | | | | | | | | | | |
|---|---|---|---|---|---|---|---|---|---|---|---|
| *Celtis zenkeri* | Cannabaceae | 26 | 22.8 | *Spondias mombin* | Anacardiaceae | 133 | 17.7 | *Blighia sapida* | Sapindaceae | 6 | 2.2 |
| *Margaritaria discoidea* | Phyllanthaceae | 48 | 22.8 | *Dracaena mannii* | Asparagaceae | 21 | 17.2 | *Citrus reticulata* | Rutaceae | 58 | 2 |
| Ficus exasperata | Moraceae | 136 | 19.7 | Bombax brevicuspe | Malvaceae | 13 | 16.7 | Antiaris toxicaria | Moraceae | 1 | 1.1 |
| Ficus mucuso | Moraceae | 31 | 19.5 | Entandrophragma angolense | Meliaceae | 52 | 16.4 | Acacia mangium | Fabaceae | 15 | 1.0 |
| Spathodea campanulata | Bignoniaceae | 29 | 18.9 | Margaritaria discoidea | Phyllanthaceae | 43 | 15.8 | Morinda lucida | Rubiaceae | 5 | 1.0 |

Our results suggest that the level of botanical knowledge among cocoa farmers is positively associated with higher tree carbon stocks (Fig 4). Farmers with a deeper understanding of tree species seem less inclined to cut them down, likely because they recognize the diverse ecological, economic, and cultural benefits these trees provide [21]. Knowledgeable farmers are more aware that remnant trees offer far more than just timber or immediate financial gain. For example, trees can serve as natural barriers against soil erosion, improve soil fertility through nitrogen fixation, and provide essential shade for cocoa plants, which helps to maintain optimal growing conditions [12], [20]. This knowledge may lead to more sustainable practices, such as selective harvesting and agroforestry, which can enhance carbon sequestration. Additionally, farmers who understand the value of trees for craft, fruit, or medicinal uses may be more likely to preserve them [29]. This is particularly important because these resources are disappearing in many landscapes due to deforestation [46, 47]. As forests are cleared for agricultural expansion or other purposes, valuable tree species that provide materials for crafts, fruits for consumption, or medicinal properties are lost, along with the cultural and economic benefits they offer. Farmers who recognize the importance of these trees are more inclined to protect them, understanding that their removal could lead to the loss of these vital resources. Ultimately, these results suggest that increased botanical knowledge could encourage practices that optimize both carbon storage and sustainable land management.

### 3.3. Carbon Hoarders : The Spontaneous

The results obtained show a significant difference in carbon sequestration capacity between planted and spontaneous trees within cocoa agroforestry systems. Spontaneous trees, which emerge through natural regeneration, exhibit a higher annual biomass accumulation rate compared to planted trees (Fig 3). It is uncommon for these two origins to be compared in terms of carbon fluxes. However, studies conducted on two Pinus species (*Pinus contorta* and *Pinus sylvestris*) in temperate forest ecosystems demonstrate that planted trees can accumulate more biomass than naturally regenerated

trees, provided they grow under optimal conditions with selected clones [48, 49]. In CAFs, such optimal conditions are not met, as trees are often wild and non-selected, whether planted or spontaneous [20]. Two main factors could explain the difference in biomass accumulation rates between planted and spontaneous trees in cocoa systems. The first factor relates to adaptation to biophysical and climatic conditions. Spontaneous trees, emerging through the process of natural regeneration [34, 50], benefit from high genetic diversity, which provides them with a greater predisposition to local conditions [51]. This diversity, shaped by natural selection, allows spontaneous trees to adapt more efficiently to biophysical constraints, such as soil type and moisture, as well as climatic stresses like droughts and heatwaves [52, 53]. The second factor concerns the quality and morphology of root systems. Planted trees often suffer from root damage during their transfer from nurseries to plantations or when they are uprooted and moved from one location to another [48]. Such damage primarily affects two types of root systems. Taproots, which are critical for anchorage and access to deep water, are frequently cut or deformed during transplantation, compromising the tree's ability to extract the resources necessary for growth [54]. Trees with damaged taproots are more vulnerable to drought and display slower growth. Superficial roots, on the other hand, may develop abnormally in nurseries due to inappropriate containers or root sleeves, leading to spiralized, compressed, or malformed roots [55]. These deformities inhibit nutrient and water absorption, thereby limiting growth and carbon sequestration capacity. In contrast, spontaneous trees, which recruit naturally on-site, develop intact root systems that are well adapted to local conditions. This promotes better root activity and, consequently, faster biomass accumulation [56].

This study highlights the socio-environmental factors influencing carbon fluxes in planted and naturally regenerated trees within cocoa agroforestry systems, identifying three main drivers: the land tenure status of the cocoa farmer, annual temperature, cocoa tree density and previous land-use (Fig 5). The carbon flux of associated trees is positively influenced by farmers who own their plots, whereas it is negatively affected by non-owner farmers. As previously shown, owner farmers tend to remove remnant trees from their fields (Fig 4), which negatively impact carbon stocks. At first glance,

the tendency of owner farmers to eliminate remnant trees while encouraging the growth of naturally regenerated ones may seem contradictory. However, the explanation lies in the differing roles and perceptions of these trees [29]. Owner farmers actively select and retain naturally regenerated trees for the services they provide, whereas remnant trees are often tolerated or endured rather than chosen [57]. This selective approach means that naturally regenerated trees receive favourable treatment, as farmers nurture them to ensure the expected benefits. Consequently, these trees exhibit normal, or even accelerated, growth due to the care provided by owner farmers. This behaviour explains the positive effect of land ownership status on the carbon flux of both planted and naturally regenerated trees.

Three additional key results shed light on the factors modulating carbon flux in cocoa agroforestry systems. (i) Carbon flux diminishes with increasing cocoa tree density, likely due to competition for resources such as light, water, and nutrients. High tree density can lead to overlapping root systems and shaded canopies, which limit the growth potential of individual trees and reduce overall carbon uptake [48]. This finding underscores the importance of optimizing tree spacing in agroforestry systems to balance productivity and carbon sequestration. (ii) Carbon flux increases with previous forest land use, likely due to higher soil fertility in areas with a history of forest cover. Forest soils often retain greater organic matter and nutrient content compared to soils converted from other land uses such as grasslands or croplands. This fertility advantage promotes vigorous growth in planted and naturally regenerated trees, enhancing their capacity to sequester carbon. These results emphasize the role of land-use history in shaping the biophysical conditions [41] that support carbon flux in cocoa agroforestry systems. (iii) As for annual temperature, negative effects on tree biomass accumulation were observed. Our results are somewhat aligned with several studies that show a negative effect of rising annual temperatures on tree biomass accumulation [27]. The temperature effect is consistent and clear: in response to rising temperatures, trees reduce their photosynthetic activity by closing their stomata to limit evapotranspiration [58, 59]. This results in a decrease in biomass production and, consequently, biomass accumulation.

## 4. Conclusions

The results of this study highlight the importance of considering remnant and spontaneous trees to optimize carbon storage and sequestration in cocoa agroforestry systems in West Africa. Among the three cohorts (planted, spontaneous, and remnant trees) evaluated for carbon stock, remnant trees support the largest stocks by far, and they play a crucial role in supporting natural regeneration for spontaneous trees. These trees are vital sources of seeds and sprouts which help maintain and enhance the natural regeneration of trees. In this way, remnant trees are fundamental for the sustainability of cocoa agroforestry systems, as they ensure the continuous recruitment of new, spontaneous trees that can contribute more effectively to carbon sequestration. In terms of biomass accumulation, spontaneous trees show a higher carbon flux than planted trees, indicating that they accumulate carbon more rapidly. Therefore, focusing on maintaining and encouraging the growth of spontaneous trees would be more beneficial for carbon sequestration than relying solely on planted trees. The role of remnant trees in sustaining this natural regeneration process is key to achieving long-term climate change mitigation in rural areas.

To optimize carbon sequestration and manage carbon stocks sustainably, it is critical to support farmers in preserving and nurturing spontaneous trees and conserve as long as possible remnant trees. This requires increasing awareness and providing training to farmers about the importance of these trees in the regeneration process. Knowledge transfer is essential for farmers to understand the value of keeping remnant trees alive, as they are not only important for biodiversity and ecosystem stability but also for enhancing carbon sequestration potential. Moreover, the ownership status of the land plays a pivotal role in encouraging farmers to let trees grow. Cocoa farmers who own their land are more likely to invest in long-term tree care and are more inclined to allow trees to grow naturally. Therefore, programs that promote land ownership and secure tenure could be crucial in motivating farmers to prioritize the preservation and growth of both remnant and spontaneous trees.

In conclusion, a comprehensive approach that combines knowledge, land tenure security, and practical support for farmers is critical to achieving effective climate change mitigation and sustainable agroforestry management in West Africa.

**Acknowledgments**

This study was conducted within the framework of the Cocoa4Future (C4F) project, which is funded by the European DeSIRA Initiative under grant agreement No. FOOD/2019/412-132 and by the French Development Agency.

**Références**


1. Gockowski J, Sonwa D. Cocoa Intensification Scenarios and Their Predicted Impact on CO2 Emissions, Biodiversity Conservation, and Rural Livelihoods in the Guinea Rain Forest of West Africa. Environ Manage [Internet]. 2011;48(2):307–21. Available from: https://doi.org/10.1007/s00267-010-9602-3
2. Harvey CA, Chacón M, Donatti CI, Garen E, Hannah L, Andrade A, et al. Climate-Smart Landscapes: Opportunities and Challenges for Integrating Adaptation and Mitigation in Tropical Agriculture. Conserv Lett. 2014;7(2):77–90.
3. Akrofi-Atitianti F, Ifejika Speranza C, Bockel L, Asare R. Assessing Climate Smart Agriculture and Its Determinants of Practice in Ghana: A Case of the Cocoa Production System. Land (Basel). 2018 Mar 1;7(1).
4. Vervuurt W, Slingerland MA, Pronk AA, Van Bussel LGJ. Modelling greenhouse gas emissions of cacao production in the Republic of Côte d'Ivoire. Agroforestry Systems [Internet]. 2022;96(2):417–34. Available from: https://doi.org/10.1007/s10457-022-00729-8
5. Läderach P, Martinez-Valle A, Schroth G, Castro N. Predicting the future climatic suitability for cocoa farming of the world's leading producer countries, Ghana and Côte d'Ivoire. Clim Change. 2013;119(3–4):841–54.
6. Schroth G, Läderach P, Martinez-Valle AI, Bunn C, Jassogne L. Vulnerability to climate change of cocoa in West Africa: Patterns, opportunities and limits to adaptation. Science of The Total Environment. 2016 Jun 15;556:231–41.
7. Middendorp RS, Vanacker V, Lambin EF. Impacts of shaded agroforestry management on carbon sequestration, biodiversity and farmers income in cocoa production landscapes. Landsc Ecol [Internet]. 2018;33(11):1953–74. Available from: https://doi.org/10.1007/s10980-018-0714-0
8. Toppo P, Professor A, Raj A, Scholar PD. Role of agroforestry in climate change mitigation. ~ 241 ~ Journal of Pharmacognosy and Phytochemistry [Internet]. 2018;7(2):241–3. Available from: http://worldagroforestry.org/newsroom/highlights/survivi
9. Dhyani SK, Ram A, Newaj R, Handa AK, Dev I. Agroforestry for carbon sequestration in tropical India. In: Carbon Management in Tropical and Sub-Tropical Terrestrial Systems. 2019.
10. Blaser WJ, Oppong J, Hart SP, Landolt J, Yeboah E, Six J. Climate-smart sustainable agriculture in low-to-intermediate shade agroforests. Nat Sustain [Internet]. 2018;1(5):234–9. Available from: http://dx.doi.org/10.1038/s41893-018-0062-8
11. Tondoh JE, Kouamé FN guessan, Martinez Guéi A, Sey B, Wowo Koné A, Gnessougou N. Ecological changes induced by full-sun cocoa farming in Côte d'Ivoire. Glob Ecol Conserv. 2015 Jan 1;3:575–95.
12. Muchane MN, Sileshi GW, Gripenberg S, Jonsson M, Pumariño L, Barrios E. Agroforestry boosts soil health in the humid and sub-humid tropics: A meta-analysis. Agric Ecosyst Environ. 2020;295(November 2019).
13. Saj S, Jagoret P, Todem Ngogue H. Carbon storage and density dynamics of associated trees in three contrasting Theobroma cacao agroforests of Central Cameroon. Agroforestry Systems. 2013;87(6):1309–20.
14. Obeng EA, Aguilar FX. Marginal effects on biodiversity, carbon sequestration and nutrient cycling of transitions from tropical forests to cacao farming systems. Vol. 89, Agroforestry Systems. Kluwer Academic Publishers; 2015. p. 19–35.
15. Dixon RK. Agroforestry systems: sources of sinks of greenhouse gases? Agroforestry Systems [Internet]. 1995;31(2):99–116. Available from: https://doi.org/10.1007/BF00711719
16. Teague M. Insights into the Cocoa and Forests Initiative: Smallholder Engagement with Certification Programs and Agroforestry. Soc Nat Resour. 2022;35(4).

17. Minang PA, Duguma LA, Bernard F, Mertz O, van Noordwijk M. Prospects for agroforestry in REDD+ landscapes in Africa. Vol. 6, Current Opinion in Environmental Sustainability. 2014.
18. Carimentrand A. Etat des lieux sur la déforestation importée et les schémas de certification de l'objectif zéro-déforestation dans la filière cacao. Rapport d'étude. Comité Scientifique et Technique Forêt/ CIRAD, février 2021, 64p Paris France.
19. Kemper, L., Sampson, G., Larrea, C., Schlatter, B., Luna, E., Dang, D., & Willer, H. The State of Sustainable Markets 2023: Statistics and Emerging Trends; 2023.
20. Kouassi AK, Zo-Bi IC, Aussenac R, Kouamé IK, Dago MR, N'guessan AE, et al. The great mistake of plantation programs in cocoa agroforests – Let's bet on natural regeneration to sustainably provide timber wood. Trees, Forests and People. 2023;12(February).
21. Sanial E, Ruf F, Louppe D, Mietton M, Hérault B. Local farmers shape ecosystem service provisioning in West African cocoa agroforests. Agroforestry Systems [Internet]. 2023;97(3):401–14. Available from: https://doi.org/10.1007/s10457-021-00723-6
22. Gamboa-Blanco EA, Dupuy JM, Portillo-Quintero CA, Caughlin T, Hernández-Stefanoni JL. Effects of successional age, plot size, and tree size on the relationship between diversity and aboveground biomass in tropical dry forests. Plant Ecol [Internet]. 2024;225(8):803–17. Available from: https://doi.org/10.1007/s11258-024-01435-4
23. Mutuo PK, Cadisch G, Albrecht A, Palm CA, Verchot L. Potential of agroforestry for carbon sequestration and mitigation of greenhouse gas emissions from soils in the tropics. Nutr Cycl Agroecosyst [Internet]. 2005;71(1):43–54. Available from: https://doi.org/10.1007/s10705-004-5285-6
24. Wagner F, Rossi V, Aubry-Kientz M, Bonal D, Dalitz H, Gliniars R, et al. Pan-tropical analysis of climate effects on seasonal tree growth. PLoS One. 2014 Mar 26;9(3).
25. Aubry-Kientz M, Rossi V, Boreux JJ, Hérault B. A joint individual-based model coupling growth and mortality reveals that tree vigor is a key component of tropical forest dynamics. Ecol Evol. 2015;5(12):2457–65.
26. Khaine I, Woo SY, Kang H, Kwak MJ, Je SM, You H, et al. Species diversity, stand structure, and species distribution across a precipitation gradient in tropical forests in Myanmar. Forests. 2017 Aug 4;8(8).
27. Chen X, Luo M, Larjavaara M. Effects of climate and plant functional types on forest aboveground biomass accumulation. Carbon Balance Manag [Internet]. 2023;18(1):1–11. Available from: https://doi.org/10.1186/s13021-023-00225-1
28. Bennett AC, Penman TD, Arndt SK, Roxburgh SH, Bennett LT. Climate more important than soils for predicting forest biomass at the continental scale. Ecography. 2020 Nov 1;43(11):1692–705.
29. Dago MR, Zo-Bi IC, Konan IK, Kouassi AK, Guei S, Jagoret P, et al. What motivates West African cocoa farmers to value trees? Taking the 4 W approach to the heart of the field. People and Nature. 2024;
30. Oyewole SO, Oyewole AL, Isola BF, Ofordu CS. Decision to Practice Agroforestry among Farmers in Ogun State of Nigeria: Implication for Climate Change Mitigation. Journal of Forests. 2022 Oct 3;9(2):39–48.
31. Wade ASI, Asase A, Hadley P, Mason J, Ofori-Frimpong K, Preece D, et al. Management strategies for maximizing carbon storage and tree species diversity in cocoa-growing landscapes. Agric Ecosyst Environ. 2010;138(3–4).
32. Batsi G, Sonwa DJ, Mangaza L, Ebuy J, Kahindo JM. Preliminary estimation of above-ground carbon storage in cocoa agroforests of Bengamisa-Yangambi forest landscape (Democratic Republic of Congo). Agroforestry Systems. 2021;0123456789.
33. Dar AA, Babu KN, Sundarapandian S, Parthasarathy N. Disentangling the response of species diversity, forest structure, and environmental drivers to aboveground biomass in the tropical forests of Western Ghats, India. Science of The Total Environment [Internet].

2024;957:177684. Available from: https://www.sciencedirect.com/science/article/pii/S0048969724078410
34. Amani BHK, N'Guessan AE, Van der Meersch V, Derroire G, Piponiot C, Elogne AGM, et al. Lessons from a regional analysis of forest recovery trajectories in West Africa. Environmental Research Letters. 2022;17(11).
35. Réjou-Méchain M, Tanguy A, Piponiot C, Chave J, Hérault B. Biomass: an R Package for Estimating Above-Ground Biomass and Its Uncertainty in Tropical Forests. Methods Ecol Evol. 2017;8(9):1163–7.
36. IPCC. Good Practice Guidance for Land Use, Land-use Change and Forestry (LULUCF). Institute for Global Environmental Strategies, Hayama, Japan 295 p, 2003.
37. Volkov I, Tovo A, Anfodillo T, Rinaldo A, Maritan A, Banavar JR. Seeing the forest for the trees through metabolic scaling. PNAS Nexus. 2022 Mar 1;1(1).
38. Carpenter B, Gelman A, Hoffman MD, Lee D, Goodrich B, Betancourt M, et al. Stan: A probabilistic programming language. J Stat Softw. 2017;76(1).
39. Andreotti F, Mao Z, Jagoret P, Speelman EN, Gary C, Saj S. Exploring management strategies to enhance the provision of ecosystem services in complex smallholder agroforestry systems. Ecol Indic [Internet]. 2018;94(July):257–65. Available from: https://doi.org/10.1016/j.ecolind.2018.06.048
40. Bastin JF, Rutishauser E, Kellner JR, Saatchi S, Pélissier R, Hérault B, et al. Pan-tropical prediction of forest structure from the largest trees. Global Ecology and Biogeography. 2018;27(11):1366–83.
41. N'Guessan AE, N'dja JK, Yao ON, Amani BHK, Gouli RGZ, Piponiot C, et al. Drivers of biomass recovery in a secondary forested landscape of West Africa. For Ecol Manage [Internet]. 2019;433(November 2018):325–31. Available from: https://doi.org/10.1016/j.foreco.2018.11.021
42. Saimun MSR, Karim MR, Sultana F, Arfin-Khan MAS. Multiple drivers of tree and soil carbon stock in the tropical forest ecosystems of Bangladesh. Trees, Forests and People. 2021 Sep 1;5:100108.
43. Kouamé IK, N'Guessan AE, Dago MR, Kouassi A, N'dja JK, Aussenac R, et al. Maximizing Tree Diversity in Cocoa Agroforestry: Taking advantage of Planted, Spontaneous, and Remnant Trees [Internet]. 2024. Available from: https://www.researchsquare.com/article/rs-4377394/v1
44. Kaba JS, Otu-Nyanteh A, Abunyewa AA. The role of shade trees in influencing farmers' adoption of cocoa agroforestry systems: Insight from semi-deciduous rain forest agroecological zone of Ghana. NJAS - Wageningen Journal of Life Sciences [Internet]. 2020;92:100332. Available from: https://doi.org/10.1016/j.njas.2020.100332
45. Baah F, Anchirinah V, Amon-Armah F. Soil fertility management practices of cocoa farmers in the Eastern Region of Ghana. Agriculture and Biology Journal of North America. 2011 Jan;2(1):173–81.
46. Ehara M, Hyakumura K, Sato R, Kurosawa K, Araya K, Sokh H, et al. Addressing Maladaptive Coping Strategies of Local Communities to Changes in Ecosystem Service Provisions Using the DPSIR Framework. Ecological Economics. 2018 Jul 1;149:226–38.
47. Ehara M, Matsuura T, Gong H, Sokh H, Leng C, Choeung HN, et al. Where do people vulnerable to deforestation live? Triaging forest conservation interventions for sustainable non-timber forest products. Land use policy. 2023 Aug 1;131.
48. Robert JA, Lindgren BS. Relationships between root form and growth, stability, and mortality in planted versus naturally regenerated lodgepole pine in north-central British Columbia. Canadian Journal of Forest Research. 2006 Oct;36(10):2642–53.
49. Abstract Agestam E, Eke PM, Johansson U. Timber quality and volume growth in naturally regenerated and planted Scats pine stands in S.W. Sweden. Studia Forestalia Suecica 204. 1998;17.

50. Amani BHK, N'Guessan AE, Derroire G, N'dja JK, Elogne AGM, Traoré K, et al. The potential of secondary forests to restore biodiversity of the lost forests in semi-deciduous West Africa. Biol Conserv. 2021;259(April).
51. Schmitt S, Tysklind N, Heuertz M, Hérault B. Selection in space and time: Individual tree growth is adapted to tropical forest gap dynamics. Mol Ecol [Internet]. 2022 Feb 13;n/a(n/a). Available from: https://doi.org/10.1111/mec.16392
52. Navarro-Cerrillo RM, Rodriguez-Vallejo C, Silveiro E, Hortal A, Palacios-Rodríguez G, Duque-Lazo J, et al. Cumulative Drought Stress Leads to a Loss of Growth Resilience and Explains Higher Mortality in Planted than in Naturally Regenerated Pinus pinaster Stands. Available from: www.mdpi.com/journal/forests
53. Mirabel A, Ouédraogo DY, Beeckman H, Delvaux C, Doucet JL, Hérault B, et al. A whole-plant functional scheme predicting the early growth of tropical tree species: evidence from 15 tree species in Central Africa. Trees - Structure and Function [Internet]. 2019;33(2):491–505. Available from: http://dx.doi.org/10.1007/s00468-018-1795-8
54. Dumroese RK, Terzaghi M, Acevedo M, Lasserre B, Scippa GS, Baggett LS, et al. Root system architecture of Pinus ponderosa three decades after copper root pruning in a container nursery. New For (Dordr). 2022 Nov 1;53(6):983–1001.
55. Haase DL, Bouzza K, Emerton L, Friday JB, Lieberg B, Aldrete A, et al. The high cost of the low-cost polybag system: A review of nursery seedling production systems. Vol. 10, Land. MDPI AG; 2021.
56. César RG, Moreno VS, Coletta GD, Chazdon RL, Ferraz SFB, De Almeida DRA, et al. Early ecological outcomes of natural regeneration and tree plantations for restoring agricultural landscapes. Ecological Applications. 2018 Mar 1;28(2):373–84.
57. Asigbaase M, Dawoe E, Lomax BH, Sjogersten S. Biomass and carbon stocks of organic and conventional cocoa agroforests, Ghana. Agric Ecosyst Environ [Internet]. 2021;306:107192. Available from: https://www.sciencedirect.com/science/article/pii/S0167880920303789
58. Khan D, Muneer MA, Nisa ZU, Shah S, Amir M, Saeed S, et al. Effect of Climatic Factors on Stem Biomass and Carbon Stock of Larix gmelinii and Betula platyphylla in Daxing'anling Mountain of Inner Mongolia, China. Advances in Meteorology. 2019;2019.
59. Schippers P, Sterck F, Vlam M, Zuidema PA. Tree growth variation in the tropical forest: understanding effects of temperature, rainfall and CO2. Glob Chang Biol [Internet]. 2015 Jul 1;21(7):2749–61. Available from: https://doi.org/10.1111/gcb.12877
60. Dunning CM, Black E, Allan RP. Later Wet Seasons with More Intense Rainfall over Africa under Future Climate Change. Available from: https://doi.org/10.1175/JCLI

Figure 1

Click here to access/download;Figure;Fig 1.tiff

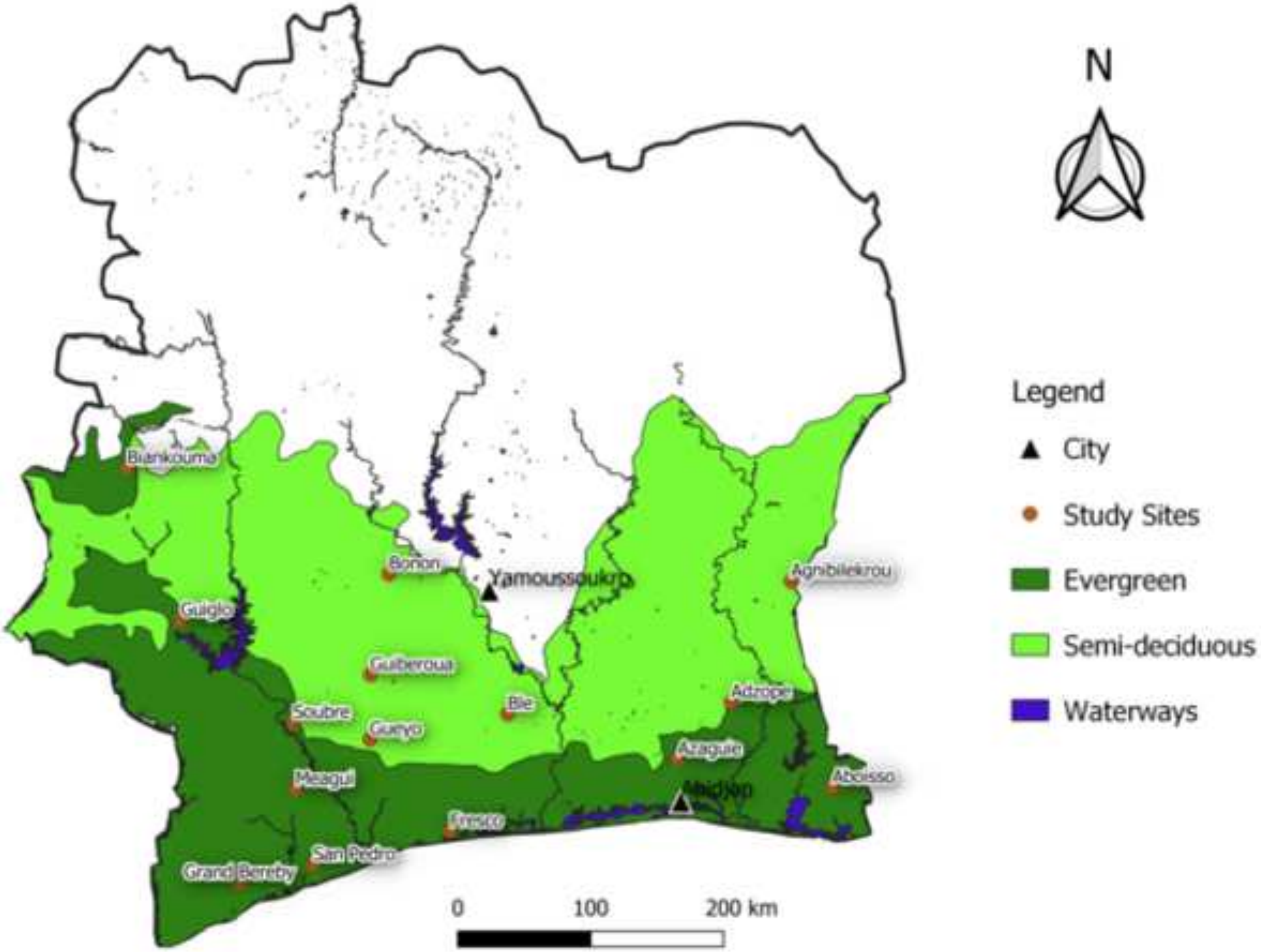

Figure 2

Click here to access/download;Figure;Fig 2.tiff

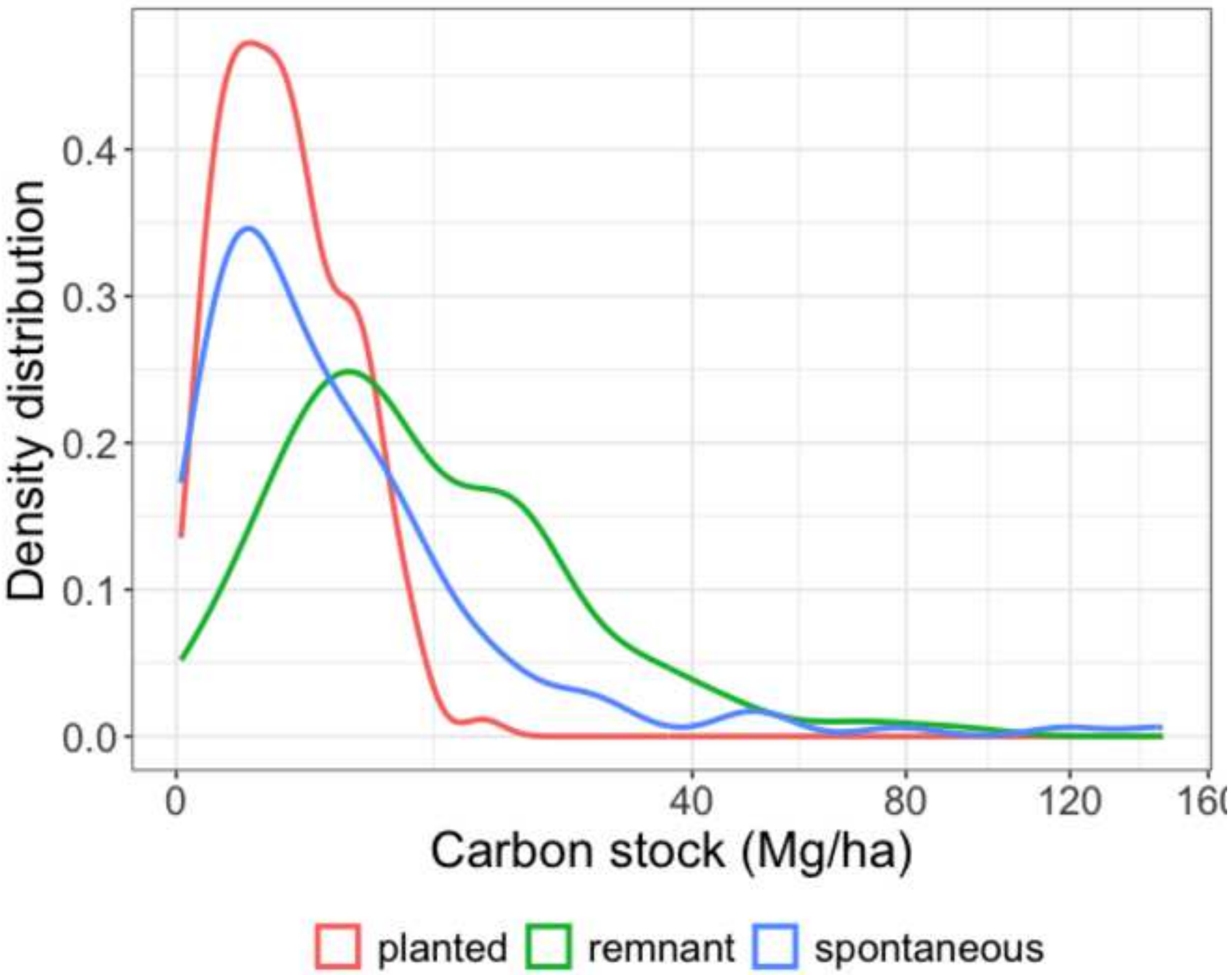

Figure 3

Click here to access/download;Figure;Fig 3.tiff

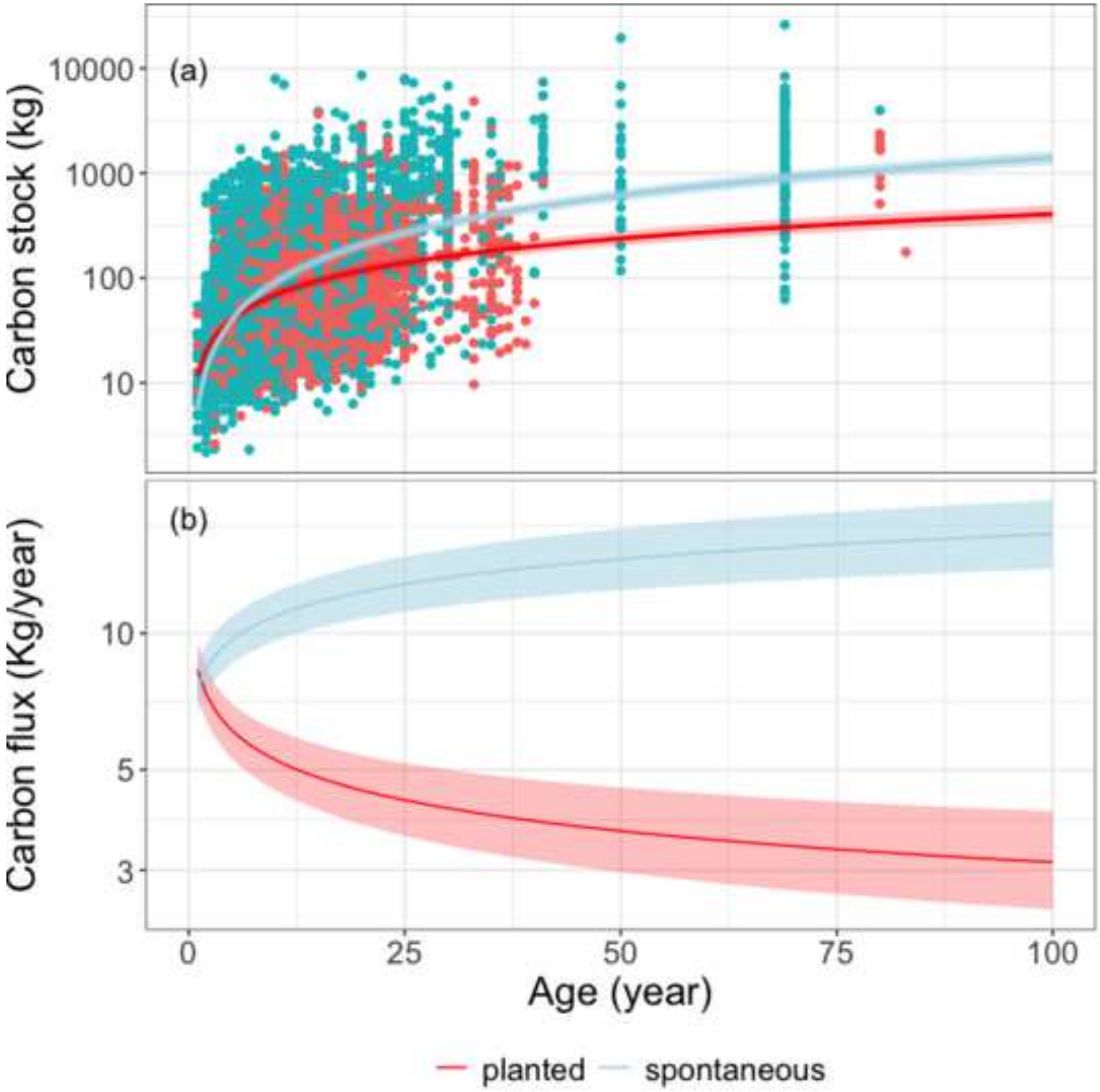

Figure 4

Click here to access/download;Figure;Fig 4.tiff

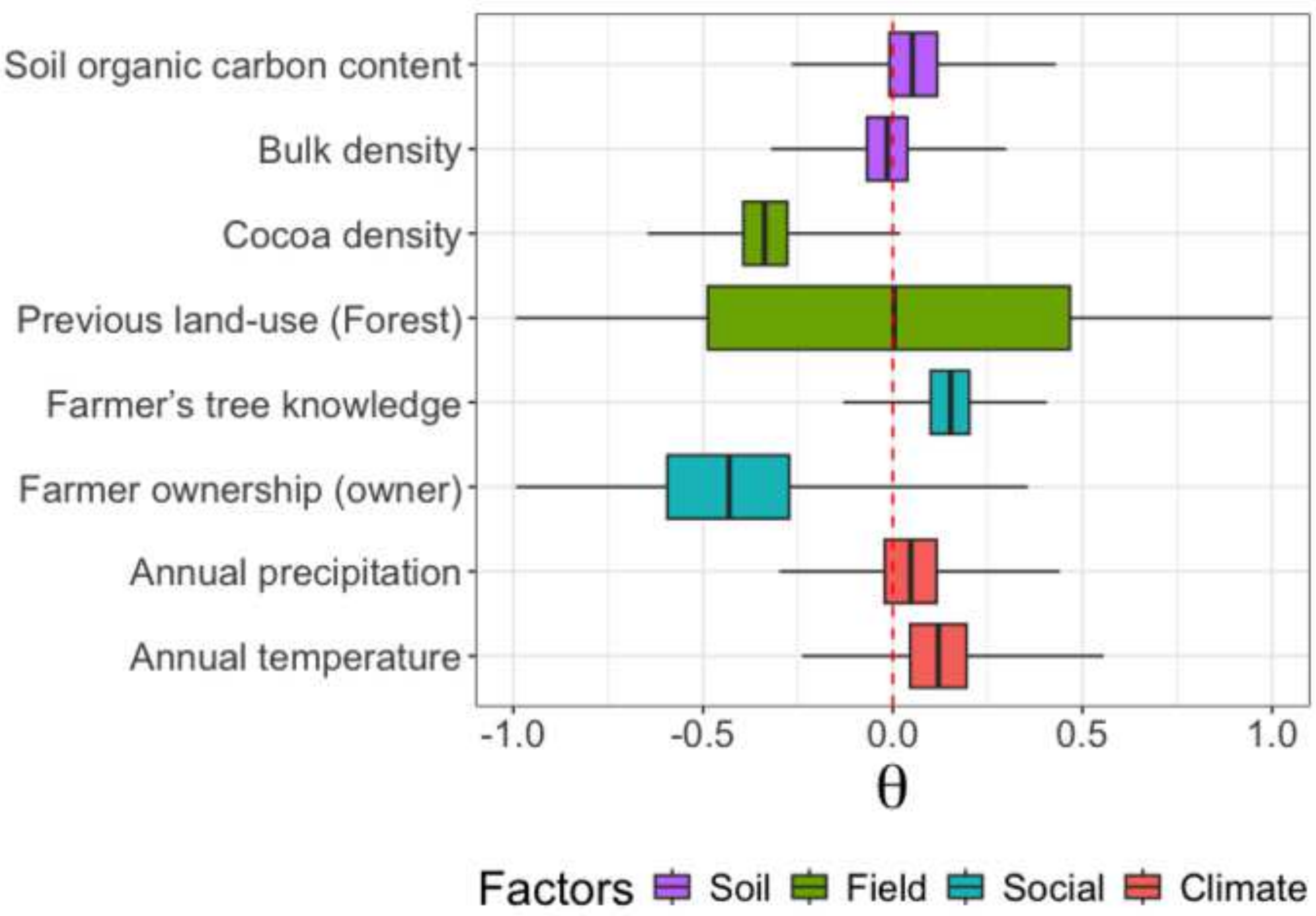

Figure 5

Click here to access/download;Figure;Fig 5.tiff

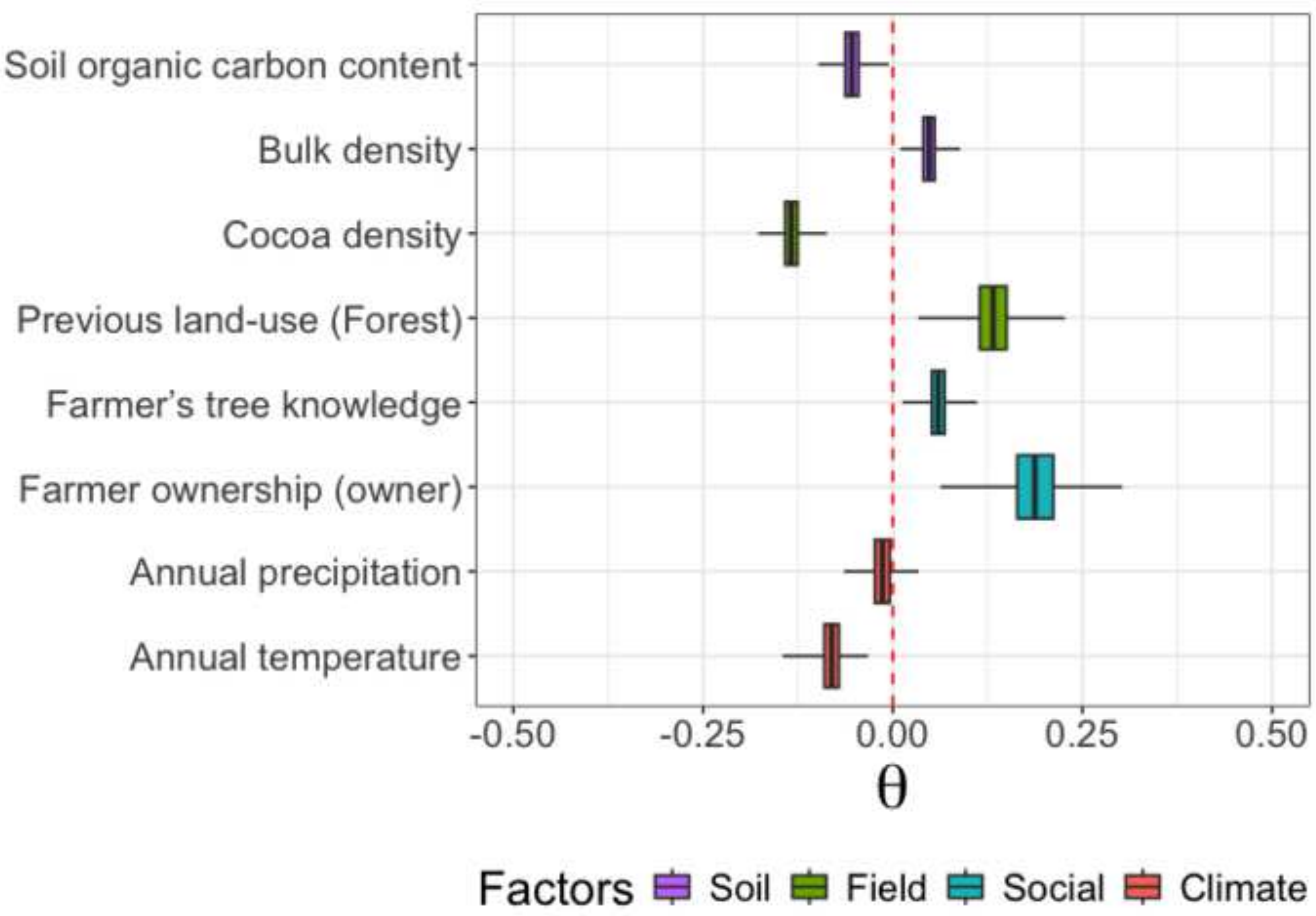

Supplement: Attachment — Submitted filename: PONE-D-25-02686_reviewer-LOA-2025.pdf [file pone.0328763.s008.pdf]
