# Supplementary material for: Unsung climate guardians: The overlooked role of remnant and spontaneous trees in carbon stocks and gains from tree growth in West African cocoa fields
Source: PLoS One. 2025 Aug 1;20(8):e0328763. doi: 10.1371/journal.pone.0328763 (PMC12316257; doi:10.1371/journal.pone.0328763)
Supplement: Attachment — Submitted filename: PlosOne_Response2-2.pdf [file pone.0328763.s010.pdf]

Contents

1 **General Comment from the Editor** 1

2 **Responses to Reviewer 4** 2

3 **Response to Reviewer 5** 6

1 General Comment from the Editor

The manuscript titled "Unsung Climate Guardians: The Overlooked Role of Remnant and Spontaneous Trees in Carbon Stocks and Gains in West African Cocoa Fields" provides a detailed review of biomass distribution among remnant, regenerating, and planted trees in cocoa agroforestry systems. This study highlights the importance of remnant trees and suggests faster carbon accumulation in regenerating trees compared to planted ones, while also offering interesting insights from the links between carbon stocks and gains and farmer knowledge and previous land use. Reviewer comments emphasize the contribution of the study, though they suggest improvements in several areas for better clarity. It is recommended to improve wording in certain lines and clarify methodological procedures, such as the calculation of tree height and the acquisition of wood density. The use of refined carbon fractions is suggested, and the growth gain analysis should be adjusted to younger trees due to the scarcity of planted trees over 30 years old. In response to previous reviews, the manuscript's update to comply with PLOS ONE's style requirements and the inclusion of an inclusivity questionnaire are appreciated. Methodological justifications and the use of Bayesian analysis have been clarified, strengthening the study's robustness. The expanded discussion on wood density and factors of natural regeneration, especially in the context of land tenure, adds significant value to the manuscript. Including a DOI for the data availability statement ensures transparency and accessibility, which are crucial for the study's reproducibility.

We would like to thank you for your constructive comments on our manuscript titled "Unsung Climate Guardians: The Overlooked Role of Remnant and Spontaneous Trees in Carbon Stocks and Fluxes in West African Cocoa Fields." Your feedback has helped us clarify certain methodological aspects and strengthen the robustness of our study.
However, we were surprised to find that the version of the manuscript sent to the reviewers was the first version we submitted, rather than the revised version we had provided. We are unsure whether this was due to a technical error or an intentional decision. This situation made it challenging to respond to some of the reviewers' comments, as several of the suggested improvements had already been addressed in the revised version. Nevertheless, we have done our best to respond clearly and transparently to each comment.

Specific Remarks and Responses

1. **Clarity of the text:** We have revised the suggested formulations to improve the clarity of the manuscript. The affected lines have been rephrased to better reflect our intentions.

2. **Methodology (tree height and wood density):** We have clarified the method for estimating tree height in the field and the procedure for acquiring wood density values from the BIOMASS package. These elements are now clearly described in the methodological section.

3. **Use of refined carbon fractions:** We have adopted the recommended carbon fractions to enhance the precision of our carbon stock estimates.

4. **Growth analysis and young trees:** We have reviewed our report on the growth gain analysis to focus on young trees due to the scarcity of planted trees over 40 years old. This modification is now specified in the corresponding section.

We hope that these modifications fully meet your expectations and remain at your disposal for any further clarification. In the following, our responses to the reviewer comments will appear in gray and the changes we made in the text in red.

2 Responses to Reviewer 4

The manuscript tittle ”Unsung Climate Guardians: The Overlooked Role of Remnant and Spontaneous Trees in Carbon Stocks and Fluxes in West African Cocoa Fields” is of general interest for agroforestry researchers and practitioners, yet in the current form, it has five main limitations.

We would like to sincerely thank the reviewer for their deep insights and constructive feedback. We carefully considered each of the points raised and made substantial efforts to better clarify the scope, methods, and interpretation of our study in the revised manuscript. Below, we summarize how we addressed the main limitations highlighted:

Misuse of the term “fluxes”
We acknowledged that our original use of the term “fluxes” was inappropriate, as our analysis does not incorporate population dynamics. In response, we adopted the more accurate term *growth gains* to reflect the individual tree-level modeling of carbon accumulation. The manuscript title and relevant sections were revised accordingly.

Gap in historical climate data
We clarified that our study does not project future climate impacts. Instead, it analyzes spatial variability in climate based on historical records 1979–2013 that are publicly available, which aligns with the main growth period of the sampled trees. This approach avoids the need to project beyond the available climate record and was explained more clearly in the revised methods section.

Assumption of a stable tree population
We clarified that our analysis does not model population-level carbon fluxes or assume a stable population. Our focus is on current carbon stocks and individual tree-level growth gains, not on future projections or dynamic carbon accounting. We revised our research questions and discussion to make this distinction explicit and avoid misunderstanding.

Interpretation of tree growth patterns (Figure 3)
We explained that while relative growth rates decline with tree age, absolute biomass accumulation can continue to increase over time, as supported by metabolic scaling theory.

We also verified that our modeling approach was appropriate and presented residual plots to demonstrate that the results are not artifacts of model bias.

Scope and focus of the discussion and conclusion

We clarified that our study does not aim to model population dynamics or elevated CO2 effects. We revised the conclusion to directly address the three initial research questions and removed speculative elements. The updated conclusion emphasizes empirically supported findings and outlines future research needs, including the importance of considering population dynamics in future studies.

Within the title the work Fluxes is not of correct use here, the authors are simply projecting carbons sequestration potential of a classes of trees on farms. Fluxes mean that you are including population dynamic in the analysis.

Thank you for your insightful comment—this is perfectly true. The term fluxes is indeed not appropriate in the context we originally used it, as our analysis does not incorporate population dynamics. Rather, our study models carbon sequestration potential at the level of individual trees. In alignment with your observation, Reviewer 5 also recommended using the term growth gains instead, and we have adopted this suggestion to improve clarity and accuracy. We have revised the manuscript accordingly. To further clarify, this part of our analysis focuses on individual tree-level carbon gains, comparing the carbon accumulation of planted versus spontaneous trees. The "growth gains" we refer to are derived directly from the carbon stock modeling performed at this individual level. We will make these points more explicit in the revised version to avoid any confusion regarding the scale and scope of our analysis.

In the text: Unsung Climate Guardians: The Overlooked Role of Remnant and Spontaneous Trees in Carbon Stocks and **Gains** in West African Cocoa Fields

The historical climate records you are using for the analysis has a GAP which might heavily affect you analysis and projection, precipitation and temperature records are available only from 1979 to 2013, so how you project the remaining climate records to account for sequestration potential of subjects trees?

Thank you for your comment. We understand the concern regarding the temporal gap in climate data. However, the most recent gridded climate dataset available to us from CHELSA (chelsa-climate.org) extends only to 2016. It is important to clarify that our study does not project future climate change impacts. Instead, we examine how spatial variability in historical climate—specifically temperature and precipitation—relates to tree growth and carbon stocks in cocoa-based agroforestry systems. This approach focuses on observed climate-growth relationships across different sites, not on modeled projections. Additionally, the reference period (1979–2013) encompasses the primary growth period for most of the trees in our dataset, making it relevant and appropriate for assessing long-term climate influences. We will clarify this methodological point in the manuscript to improve transparency.

In the text: These data were used to assess the effect of spatial climate variability—not future projections—on tree growth and carbon stocks in cocoa agroforestry systems.

The analysis and assumption the authors used in the study is no entirely correct, the authors are analysis and projecting on farm tree population as "Stable" population, no changes are foreseen, but this is incorrect, trees on farms (specially the spontaneous) experience recruitment rates, mortality rate and harvest rates, none of this aspect are considered in the analysis not even in the discussion section, therefore the projection of carbon sequestration potential are not valid. The authors need to include the tree management aspect into the analysis/discussion.

Thank you for your thoughtful comment. You're absolutely right that understanding carbon fluxes over time requires accounting for tree population dynamics, including recruitment,

mortality, and harvesting. However, we would like to clarify that our study does not involve projections or long-term modeling of carbon sequestration potential. As noted in response to your earlier comment, our analysis is conducted at the individual tree level rather than the population level. We do not assume a ”stable” tree population, nor do we attempt to predict future carbon stocks. Instead, our focus is on comparing the current carbon past sequestration of planted versus spontaneous trees under varying climate conditions in space. Since our objective is not to forecast future carbon accumulation, tree management dynamics—while important in broader carbon accounting—fall outside the scope of this specific analysis. Nonetheless, we agree that this distinction should be made clearer in the manuscript and we have revised the research questions and the discussion section accordingly to avoid any misunderstanding.
In the research questions: (1) At the cocoa field level, what are the carbon stocks of planted, spontaneous, and remnant trees? (2) At the individual tree level, what is the effect of origins (planted, spontaneous) on the carbon gains from tree growth? (3) What are the key socio-environmental factors influencing carbon stocks (at the field level) and gains (at the tree level) in these systems?
In the text: This difference reflects variations of past tree growth in relation to spatial climate variability, based on individual trees sampled across sites.

Results from Figure 3 are quite confusing, growth patter of trees usually follow a S shape curve which means tree growth rates decreases overtime until a plateau is reached, therefore trees sequestration potential decreases overtime, however, figure 3 show low or even decreasing sequestration rate, quite the opposite of spontaneous trees, this findings require eighter a review of the growth model used (the allometric equations that is useful for the dbh range of inventory trees) for this particular tree class or a well references discussion quite far for ”nursery issues”.
Thank you for this important observation. You are correct that diameter growth typically follows an S-shaped curve over time, with growth rates declining as trees mature. However, when it comes to biomass accumulation, the dynamics are different. According to metabolic scaling theory (e.g., Enquist et al., 1999; West et al., 1997), biomass growth tends to increase with tree size in the early and middle stages of development, because larger trees have more leaf area and therefore higher photosynthetic capacity, leading to greater absolute carbon accumulation—even if diameter growth slows. In other words, while relative growth rates (e.g., % increase per year) decrease over time, absolute biomass gains continue to increase for several decades. Additionally, we carefully checked the validity of the model used. We provide below the plot of the model residuals, and found no systematic trends that would indicate bias related to tree size or class. This suggests that the observed differences are not artifacts of model selection, but rather reflect real pattern in the data.

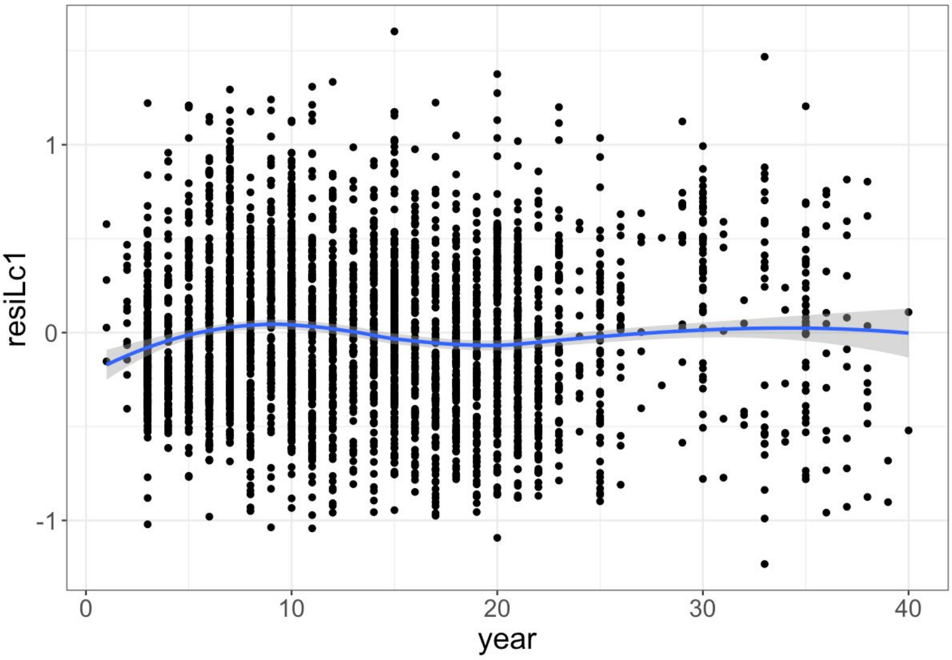

Figure 1: Residuals of the Carbon Model

The discussion is out of focus, there is a lack of discussion regarding population dynamics of the guardian trees in cacao farms as well as the potential effects of elevated C02 on overall tree growth. The conclusion is quite speculative, in covers topics such as seed dispersal, natural regeneration, farmers training, etc and does not answers the three initial research questions. Conclusion is overall redundant, speculative and vague.

Thank you for your detailed feedback. We respectfully disagree with the assertion that the discussion is out of focus. Our study does not aim to address population dynamics or simulate the effects of elevated CO on tree growth, as our methodological approach is observational rather than predictive. Specifically, we focus on quantifying current tree carbon stocks at the plot level and assessing individual tree-level gains based on historical growth—rather than projecting future dynamics or responses to climate variables. The structure of the discussion aligns with the research questions analyzed in our study. In response to earlier reviewer feedback, we expanded our discussion of farmers' training and land use history, as these were identified as key explanatory factors influencing tree carbon outcomes and were deemed critical by previous reviewers. Regarding the conclusion, we acknowledge the concern and have revised it to more clearly and directly respond to the three initial research questions, removing speculative elements and ensuring better alignment with our empirical findings. We appreciate your comments and thank you for your understanding.

The new conclusion with explicit structure related to the 3 research questions:

This study provides a detailed assessment of carbon dynamics in cocoa agroforestry systems by examining carbon stocks and tree-level carbon gains, while also identifying the key socio-environmental factors that influence these outcomes.

(1) At the field level, we found that remnant trees store the largest share of aboveground carbon, far surpassing that of planted and spontaneous trees. These legacy trees represent a critical carbon reservoir within cocoa fields and are essential for maintaining structural diversity and supporting regeneration processes. Spontaneous trees, while contributing less to total field-level carbon stocks, add meaningful amounts and play an important complementary role in sustaining agroforestry resilience.

(2) At the individual tree level, we observed that spontaneous trees accumulate more carbon from growth over time than planted trees, indicating their higher carbon sequestration efficiency. This suggests that encouraging the growth and survival of spontaneously established trees could be more effective for increasing biomass accumulation and carbon sequestration in the medium term than relying solely on planted individuals.
(3) Regarding socio-environmental drivers, two key factors emerged: farmers' training and land tenure security. Farmers with greater botanical knowledge were more likely to preserve remnant trees and allow spontaneous trees to grow. Likewise, land ownership significantly influenced carbon outcomes, as landowners were more inclined to invest in long-term tree management practices, including the retention and nurturing of naturally regenerating trees.
Together, these findings emphasize that optimizing carbon storage in cocoa agroforestry systems requires more than just planting efforts. Instead, it demands an integrated strategy that values and supports remnant and spontaneous trees, enhances farmers' awareness and technical capacity, and ensures secure land tenure. These elements are critical to enabling rural communities in West Africa to contribute meaningfully to climate change mitigation while sustaining productive agroforestry landscapes. Additionally, future research should assess the carbon sequestration capacity of both planted and spontaneous trees at the population level, taking into account tree mortality and population dynamics. Such studies would contribute to a more comprehensive understanding of the carbon balance in cocoa agroforestry systems and provide deeper insights into their long-term role in climate change mitigation.

3 Response to Reviewer 5

This manuscript surveys trees in cocoa agroforestry and partitions biomass between remnant, regenerating and planted trees informed by surveys with farmers. This reinforces the importance of remnant trees, but also supports faster carbon accumulation from regenerating rather than planted trees. There are also interesting insights into the effects of farmer knowledge and prior land-use. This is an interesting contribution, although I have a number of points that could do with more clarity. I could not see a response to reviewers, so apologise if I have suggested something that was rebutted previously.
Thank you for your interest in this research and for the valuable comments you have provided.

Line 37-38 – Awkward phrasing – better as "Carbon stocks were higher where farmers had greater knowledge and where there was previous forest land use"?
Rephrased.
In the text: Carbon stocks rose with informed farmers and former forest use, but declined with higher cocoa density.

Throughout – I'd personally talk about passively regenerating trees (or naturally regenerating trees) rather than spontaneous trees, as I find the latter wording a bit strange, but it is clearly defined so up to the authors.
Thank you for the suggestion. We appreciate your perspective on terminology. While we understand that "naturally regenerating" or "passively regenerating" trees may sound more conventional, we have chosen to retain the term "spontaneous trees" for consistency with our previous publications, where it is clearly defined and used throughout. Maintaining this terminology allows for continuity across our body of work and ensures alignment with how we've classified tree origins in related studies.

82 – slow growth rather than stunt growth, as I think this is saying low precipitation slows growth rates rather than diminishes tree final size.
Agreed

Line 128 – How many ha were surveyed?
Our survey was done across 240.5 ha.
In the text: The study plots, covered a total area of 240.5 ha, vary in size from 0.3 to 5 hectares and serve as the sampling units, corresponding to the management units of the farmers.

Line 178 – Probably not worth redoing all the analysis for, but would be better to use one of the refined carbon fractions from Thomas and Martin 2018 rather than the IPCC default.
Agreed—thank you for the suggestion. We've updated the carbon estimates throughout the text using the refined fraction from Thomas and Martin (2018). While this change does not affect the relationships between explanatory variables, it provides a more accurate assessment of carbon stocks, which we appreciate.

Line 178 – Which procedure was used to estimate tree height? The environmental stress parameter? Also define how wood density was obtained (taxonomic lookup through getWD I presume, if so state matches at species and genus level, and if family or plot-level values were used for taxa not matched at genus level).
Thank you for your comment. Tree height was estimated in the field using a Suunto dendrometer. Because we directly measured tree height, it was not necessary to estimate or define the environmental stress parameter (E) that is needed in BIOMASS to overcome the absence of height values. Indeed, E is only required when height needs to be modeled from diameter and environmental conditions only. Wood density was retrieved using the getWoodDensity() function from the BIOMASS package. Values were matched at the species level when available, and at the genus level when species-level data were not present. We did not use family- or plot-level averages, as genus-level matches were available for all remaining cases.
In the text: The computeAGB() function uses the pantropical allometric equation of Chave [38] to estimate the aboveground biomass, incorporating wood density values obtained via the getWoodDensity() func-tion—matched at the species level when available, or at the genus level otherwise.

Line 181 – This isn't net carbon flux, it is carbon flux due to growth, and potentially would be better named as carbon gains or similar. Within a plot, there will be carbon fluxes in due to growth and out due to mortality, including unobserved growth of trees that have now died. What is looked at here is one part of this – fluxes in due to growth of the cohort of surviving trees. This needs to be more tightly defined.
Completely agreed—thank you for this important clarification. We have revised the terminology throughout the manuscript, replacing "carbon flux" with "carbon gains" to more accurately reflect that our estimates capture only the carbon accrued through the growth of surviving trees, and do not account for losses due to mortality or unobserved growth of now-dead individuals. This refinement improves the precision of our interpretation and presentation.

Line 195 – It would be helpful to explain (in words rather than an equation) what coefficients are free to vary with what in this model. If I understand right, there is a power law effect of age which varies between cohorts, and observed biomass separately varies with a set of socio-ecological variables. So age-specific effects can vary between plots, but do not explicitly interact with socio-ecological factors? Also, why are age power-law effects allowed to vary if they are

constrained by metabolic and geometric scaling? Is it because these are size-scaling factors, and you are capturing the varying age-size relationship?
Thank you for your thoughtful and detailed comments. We have clarified the model structure in the revised manuscript and address your points below. In our model, carbon gains (Cf) for individual trees are estimated using a log-normal distribution, where the expected value is a function of:

- A cohort-specific power-law effect of tree age, allowing the relationship between age and carbon gain to vary between planted, spontaneous, and remnant trees;
- A species-level coefficient capturing inherent differences in carbon gain across species;
- And a set of socio-ecological covariates at the plot level, which influence carbon gain independently of age or cohort.

The effects of age and socio-ecological variables are multiplicative, and while both contribute to explaining variation in carbon gains, we do not include interactions between them in the current model. That is, the age effect is allowed to vary across tree origin cohorts but does not explicitly depend on the socio-ecological context.
Regarding your question on the rationale for allowing the age power-law exponent to vary between cohorts: although metabolic and geometric scaling theories suggest that biological processes often follow power-law relationships, they are more informative about the functional form of models than about fixed parameter values. In reality, the exponent may vary due to a range of intrinsic factors (e.g., species traits, cohort origin) and extrinsic factors (e.g., environmental stress, land-use history, management). By allowing the age exponent to vary by cohort, we aim to empirically capture these differences in growth dynamics—particularly since the different cohorts experience distinct ecological conditions and management histories. This approach preserves the theoretical shape inspired by metabolic theory while remaining flexible and empirically grounded.
We hope this clarification helps to better convey both the logic and flexibility of our modeling strategy. Text has been updated accordingly

Line 207-208 – While there are advantages with this modelling framework, the biggest uncertainty is the likely large errors in farmer estimates of tree age. This is not accounted for in this modelling framework nor a simple regression, and it needs to be clearly stated that there is a big uncertainty which is not accounted for. I know this is talked about in the discussion, but mention here as well.
Thank you for your observation. We are in agreement with you and have made adjustments in this section, which you can see below.
In the text: However, it is important to note a key limitation of the modelling framework: while we propa-gate uncertainty related to model parameters and observed covariates, we are not able to account for potential errors in farmer-reported tree age, which likely introduces additional, unquantified uncertain-ty into our estimates of carbon gains.

Line215-224 – I'd find it helpful to have some boxplots (e.g. size class, wood density) between the groups.
Agreed. Distributions of DBH, Height and Wood densities are now included in Appendix 2 - 3 - 4.

Line 240 – Watch use of European decimal (, vs .).
Thank you. It is a mistake, we don't use European decimal. We use U.S. decimal (.).

Line 253 – Define what the plus minus values are showing.

Thank you for the observation. The "±" notation refers to the posterior mean and its associated standard deviation, representing uncertainty under the Bayesian framework. As this is a standard convention in statistical reporting, we did not explicitly define it in the text. However, we are happy to add a brief clarification if the editor feels it would enhance clarity for readers.

Line 294-301 – I think the discussion of remnant trees can be improved. It should distinguish between tree species identify (e.g. are dense wooded species left as they are hard to cut [no according to non-significant differences in wood density]) and the more obvious they store more carbon because they are old big trees rather than young ones.

Thank you for your feedback. We have revised this section of the discussion to enhance its clarity.

In the text: Our results show that carbon stock in cocoa agroforestry systems (CAFs) is strongly influ-enced by the presence of remnant trees (Fig 2). This finding is consistent with previous studies (e.g. Andreotti [41], Bastin [42], and Sanial [21]), which have also reported that remnant trees contribute disproportionately to aboveground carbon storage compared to planted or spontaneously regenerated cohorts. Remnant trees are typically large, mature individuals that were spared during land clearing—either intentionally or due to logistical constraints [43]. They often include species such as Milicia excelsa (Welw.) Benth, Alstonia boonei De Wild, Amphimas pterocarpoides Harms and Petersian-thus macrocarpus (P. Beauv.) Liben (Table 3), which have high carbon storage because of their large diameter and height [21]. As expected, tree size directly determines carbon stock, with taller and thick-er trees storing significantly more carbon [22]. While it could been hypothesized that farmers may selectively leave certain species—such as those with high wood density—because they are difficult to cut, our results show no significant differences in wood density among cohorts (Appendix 4). In ad-dition to size, species diversity may also contribute to higher carbon stocks. The remnant cohort exhibit the highest species among the three groups [21, 43], and previous research has shown that great-er tree diversity is often associated with increased biomass and carbon storage [22, 32, 42]. Thus, the value of remnant trees lies not only in their individual size but also in the structural and ecological di-versity they bring to these landscapes.

Figure 3. There are very few planted trees more than 30 years old, so maybe restrict the analysis of growth gains to the younger trees.

Indeed, most planted trees are less than forty years old, and this also applies to spontaneous trees. We do restrict the analysis of carbon gains to trees less than 40 years old and adjust the text accordingly.

Furthermore, because very few trees exceed 40 years of age, the analysis of growth-derived carbon gains is effectively restricted to younger trees, ensuring more reliable age estimates and minimizing uncertainty related to farmer recall.

Figure 4/5. Caution is needed when inferring causality about farmer knowledge. Could farmers with more diverse agroforests (or living closer to forests) know more tree species?

Thank you for this important observation. We agree that inferring causality regarding farmer knowledge is challenging. It is indeed difficult to fully control for factors such as proximity to forests or prior exposure to diverse landscapes—especially in a context where farmer populations are highly dynamic due to migration and resettlement. We have now added a sentence in the discussion to acknowledge this limitation and to clarify that our findings reflect associations rather than causal relationships.

In the text: We also recognize a limitation regarding the interpretation of farmer botanical knowledge. While our findings show a positive association between farmer knowledge and higher tree carbon stocks and gains, causality may be questioned. Landscapes with higher tree diversity—such as former forest areas—may naturally promote greater knowledge through prolonged exposure, and migration patterns may further complicate the relationship.
